# split-intein Gal4 provides intersectional genetic labeling that is repressible by Gal80

**DOI:** 10.1073/pnas.2304730120

**Published:** 2023-06-05

**Authors:** Ben Ewen-Campen, Haojiang Luan, Jun Xu, Rohit Singh, Neha Joshi, Tanuj Thakkar, Bonnie Berger, Benjamin H. White, Norbert Perrimon

**Affiliations:** ^a^Department of Genetics, Blavatnik Institute, Harvard Medical School, Boston, MA 02115; ^b^Laboratory of Molecular Biology, National Institute of Mental Health, NIH, Bethesda, MD 20892; ^c^CAS Key Laboratory of Insect Developmental and Evolutionary Biology, CAS Center for Excellence in Molecular Plant Sciences, Chinese Academy of Sciences, Shanghai 200032, China; ^d^Computer Science and Artificial Intelligence Laboratory, Massachusetts Institute of Technology, Cambridge, MA 02139; ^e^Department of Mathematics, Massachusetts Institute of Technology, Cambridge, MA 02143; ^f^HHMI, Boston, MA 02115

**Keywords:** *Drosophila*, split-Gal4, intersectional genetics, single-cell transcriptomics

## Abstract

The split-Gal4 system allows for intersectional genetic labeling of highly specific cell types and tissues in *Drosophila*. However, the existing split-Gal4 system, unlike the standard Gal4 system, cannot be repressed by Gal80, and therefore cannot be controlled temporally. This lack of temporal control precludes split-Gal4 experiments in which a genetic manipulation must be restricted to specific timepoints. Here, we describe a split-Gal4 system based on a self-excising split-intein, which drives transgene expression as strongly as the current split-Gal4 system and Gal4 reagents, yet which is repressible by Gal80. We demonstrate the potent inducibility of “split-intein Gal4” in vivo using both fluorescent reporters and via reversible tumor induction in the gut. Further, we show that our split-intein Gal4 can be extended to the drug-inducible GeneSwitch system, providing an independent method for intersectional labeling with inducible control. We also show that the split-intein Gal4 system can be used to generate highly cell type–specific genetic drivers based on in silico predictions generated by single-cell RNAseq (scRNAseq) datasets, and we describe an algorithm (“Two Against Background” or TAB) to predict cluster-specific gene pairs across multiple tissue-specific scRNA datasets. We provide a plasmid toolkit to efficiently create split-intein Gal4 drivers based on either CRISPR knock-ins to target genes or using enhancer fragments. Altogether, the split-intein Gal4 system allows for the creation of highly specific intersectional genetic drivers that are inducible/repressible.

## Significance

The split-Gal4 system allows *Drosophila *researchers to drive transgene expression with extraordinary cell type specificity. However, the existing split-Gal4 system cannot be controlled temporally, and therefore cannot be applied to many important areas of research. Here, we present a split-Gal4 system based on a self-excising split-intein, which is controllable by Gal80, as well as a related drug-inducible split GeneSwitch system. This approach can both leverage and inform single-cell RNAseq datasets, and we introduce an algorithm to identify pairs of genes that precisely and narrowly mark a desired cell cluster. Our split-intein Gal4 system will be of value to the *Drosophila *research community, and allow for the creation of highly specific genetic drivers that are also inducible/repressible.

The ability to restrict transgene expression to specific, genetically defined cell types using binary expression systems such as Gal4/UAS, LexA/LexAOP, and QF/QUAS has profoundly transformed *Drosophila* research (1–3). In particular, the Gal4 system has been deployed extraordinarily effectively, with thousands of Gal4 drivers available in *Drosophila* resource centers. However, the lack of tissue- and cell-type specificity of many Gal4 drivers remains a drawback. This is especially true for certain areas of research. For example, studies of interorgan communication in which a Gal4-driven manipulation is performed in one tissue and the effects are measured in a distant tissue must take special care to avoid the confounding effects of Gal4 expression outside of the intended tissue (4). Similarly, many neurobiological studies require Gal4 expression to be limited to one, or very few, transcriptionally defined neuron, which is not generally possible using standard Gal4 drivers, even when driven by 2 to 3 kb genomic enhancer fragments (5–7).

**Fig. 1.**
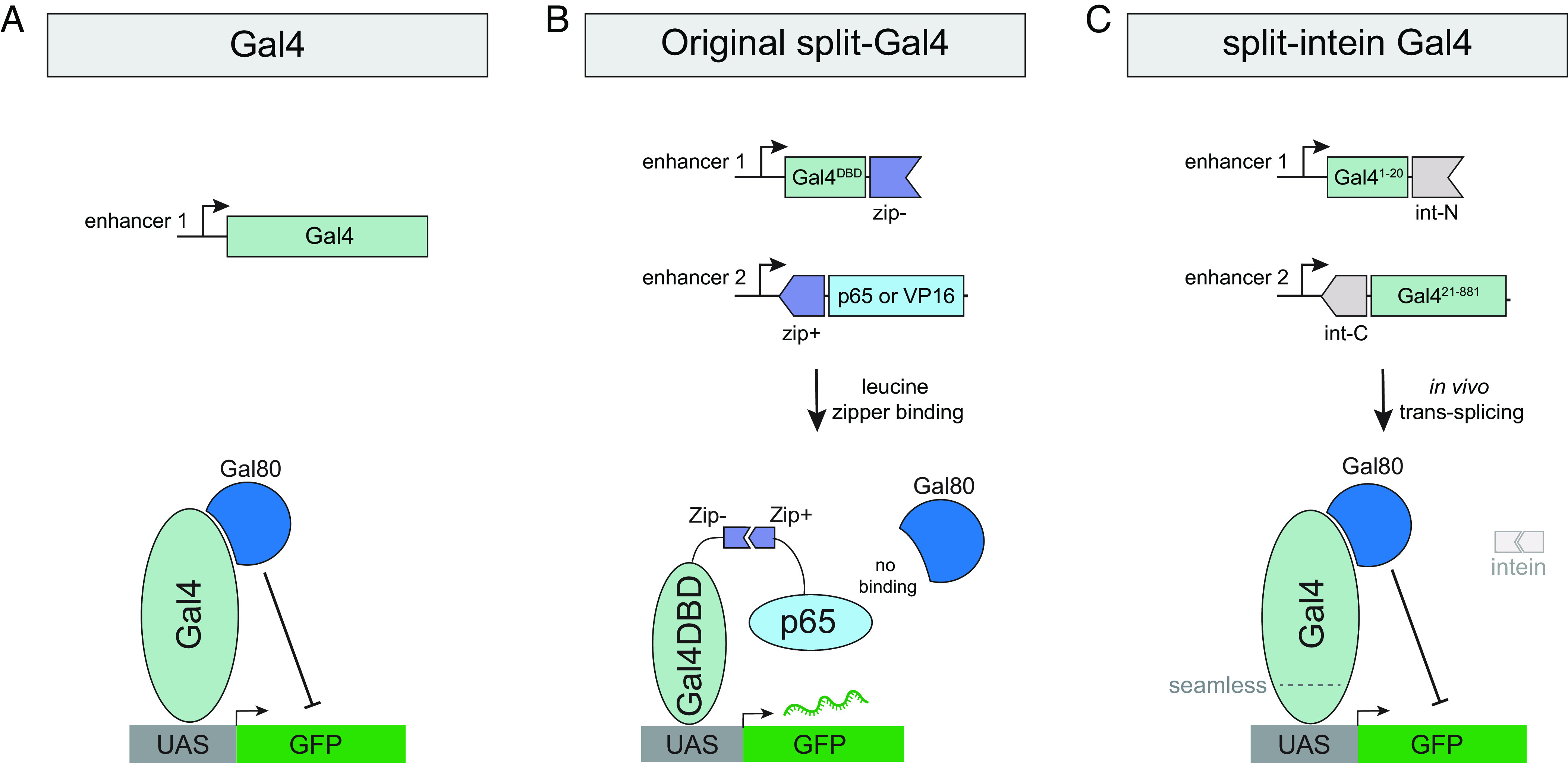
Schematic comparison of Gal4, split-Gal4, and split-intein Gal4. (*A*) The Gal4 transcription factor binds to UAS sequences to drive transcription and can be repressed by the binding of Gal80. Gal4 is drawn here as a monomer, but functions as a dimer in vivo. (*B*) In the original split-Gal4 system, the Gal4DBD and a strong transcriptional activator (VP16 or p65) are each driven by separate enhancers and reconstituted in cells by leucine zipper domains. Gal80 cannot bind or repress the split-Gal4 complex. (*C*) In the split-intein Gal4 system, two fragments of the Gal4 protein, each flanked by a split-intein, are independently driven by separate enhancers and seamlessly transspliced to reconstitute a functional, wild-type Gal4 protein, which can be repressed by Gal80.

The split-Gal4 system was developed to overcome the issue of limited cell-type specificity, by restricting transgene expression to those cells that coexpress two independent enhancers, a strategy termed “intersectional genetic labeling” (8, 9). In split-Gal4, the N-terminal 147 amino acids of Gal4, which includes its DNA-binding domain (Gal4DBD) (10) and its dimerization domain (11), is expressed under the control of one enhancer, while a potent transcriptional activator domain (AD) from either VP16 or p65 is expressed under the control of a second enhancer (Fig. 1) (8, 12). The Gal4DBD and the VP16/p65 activation domains are each flanked by a leucine zipper domain, which heterodimerize in any cell expressing both components, and reconstitute a functional Gal4-like transcription factor (8, 9). The split-Gal4 system has been successfully used to build thousands of exquisitely specific genetic drivers, especially in the *Drosophila* nervous system where split-Gal4 lines are now routinely utilized to drive expression in a single pair of neurons (6, 7), and in the adult gut, where thousands of split-Gal4 lines have been characterized (13, 14). The ability to create split-Gal4 lines that are specifically expressed in the same patterns as genes of interest using “trojan exons” or other knock-in strategies has further augmented the power of the split-Gal4 method (15). This capability has particular promise in permitting the construction of genetic driver lines that target transcriptionally distinct clusters identified via scRNAseq studies (16). For such clusters, the intersection of at least two genetic markers is typically necessary to uniquely identify specific clusters.

**Fig. 2.**
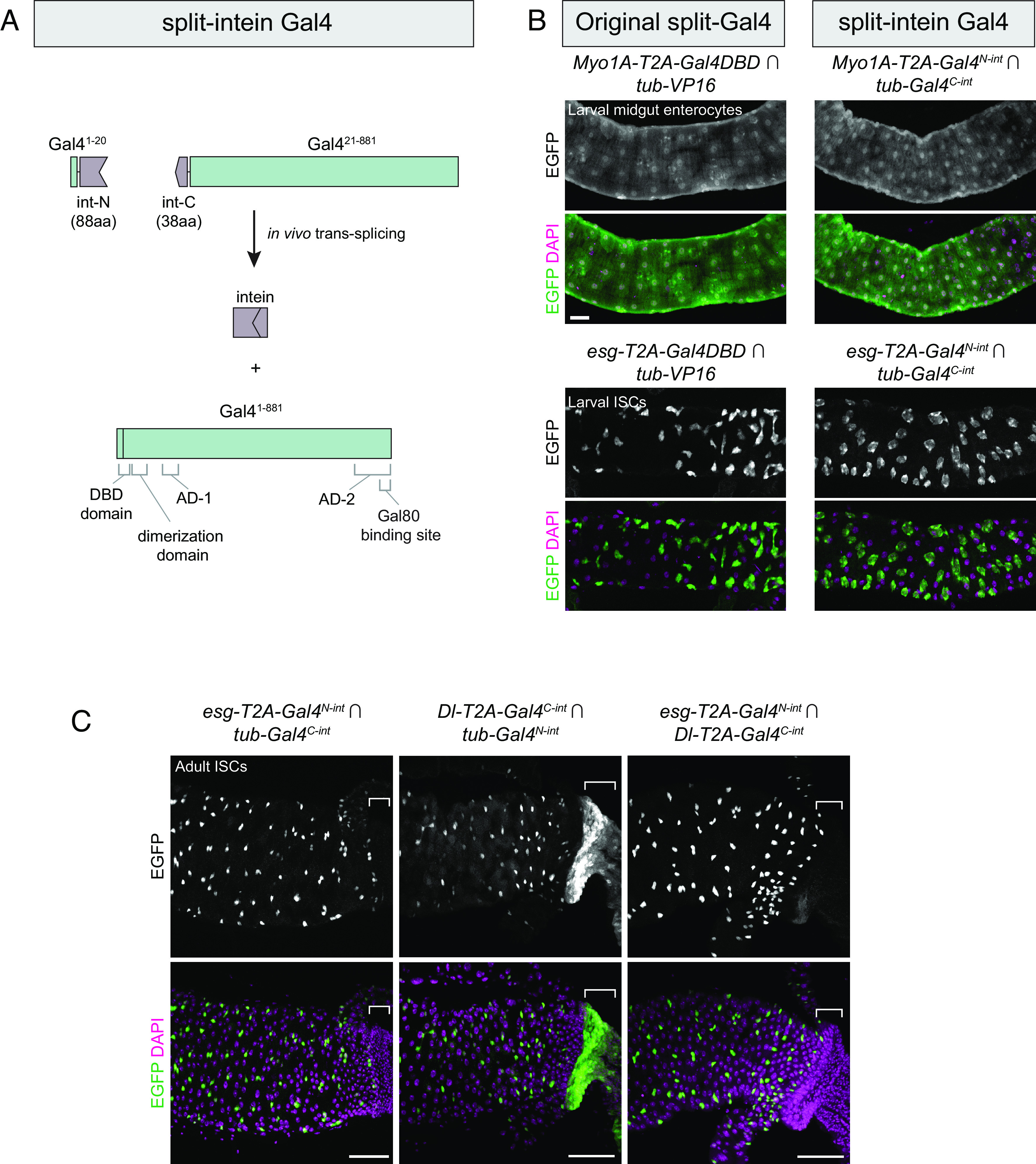
Split-intein Gal4 system drives intersectional expression at levels indistinguishable from split-Gal4. (*A*) Schematic diagram of the split-intein Gal4 system. Gal4 and gp-41 are drawn to scale, illustrating the N-terminal DNA-binding domain (DBD) and dimerization domain, and the overlap of the second activation domain (AD-2) with the Gal80-binding site. (*B*) Components for original split-Gal4 (*Left*) or split-intein Gal4 (*Right*) were knocked into two gut cell-type markers: *Myo1A,* which labels enterocytes (ECs), and *esg,* which labels intestinal stem cells (ISCs). These knock-in lines were crossed to ubiquitously expressed tester lines to visualize their full expression pattern. (*C*) Intersectional labeling of midgut intestinal stem cells using *esg* ∩ *Dl* split-intein Gal4 knock-in lines. Brackets indicate expression in the anterior hindgut which is driven by *Dl* but not *esg*, which is absent in their intersection. Anterior is to the left. (Scale bars, 50 µm.)

But while the split-Gal4 system has effectively solved the problem of restricting expression in anatomical space, the existing split-Gal4 system cannot be controlled in time. This is in contrast to standard Gal4 drivers, which can be temporally controlled using a temperature-sensitive variant of the Gal80 repressor (Gal80^ts^) (17). By shifting between a permissive temperature (18 °C), where Gal80^ts^ represses Gal4 expression, and a restrictive temperature (29 °C), where Gal80^ts^ is inactivated and Gal4 becomes active, researchers can restrict genetic manipulations to specific time periods or developmental stages. By contrast to the standard Gal4 system, the split-Gal4 system is completely insensitive to the Gal80 repressor (9). This is because the region of Gal4 that is bound by Gal80, the C-terminal 30 amino acids (18), falls squarely within the Gal4 AD domain, which is replaced in existing split-Gal4 implementations with either VP16 or p65 in order to drive sufficiently high levels of expression (Figs. 1 and 2*A*). Thus, the Gal4DBD-VP16 or Gal4DBD-p65 protein complexes do not contain any binding site for Gal80 and therefore cannot be repressed (Fig. 1).

There is thus a clear need for an intersectional labeling system that is repressible by Gal80^ts^ or otherwise inducible. Here, we describe two such systems. The first we term “split-intein Gal4.” This system combines the enhanced spatial control offered by split-Gal4 with the ability to strictly limit genetic manipulations to specific periods of time using existing Gal80^ts^ reagents. We demonstrate that split-intein Gal4 drives UAS transgenes at expression levels that are indistinguishable from existing split-Gal4 and Gal4 reagents and that it can be repressed by standard Gal80 reagents. The second system is a closely related drug-inducible GeneSwitch technique (“split-intein GeneSwitch”), which provides an alternative means to induce intersectional genetic labeling using a drug rather than a temperature shift. Finally, we demonstrate that the split-intein Gal4 system can be effectively used with scRNAseq datasets to generate split-intein Gal4 driver lines. To facilitate production of such lines, we present an algorithm to select gene pairs with low levels of predicted coexpression outside the cluster of interest. Finally, we provide a simple cloning and transgenesis workflow that can be used to generate large numbers of split-intein-Gal4 lines, either via CRISPR-based knock-in or using enhancer fragments.

## Results

### Designing a Split-Gal4 Technique That Can Be Repressed by Gal80.

We wished to create an inducible/repressible intersectional labeling technique that can be controlled temporally. We focused our efforts on modifying the highly effective split-Gal4 concept, to make a split system that did not rely on leucine zipper heterodimerization and that incorporated the native Gal4 activation domain, thus rendering it sensitive to the Gal80^ts^ repressor. We devised two independent strategies, which we refer to as “split-intein Gal4” and “NanoTag split-Gal4.”

#### Split-intein Gal4.

Native split-inteins consist of N- and C-terminal peptides that are fused to proteins encoded at separate genomic loci. Upon translation, these peptides associate with one another, self-excise, and seamlessly trans-splice the two adjacent polypeptide chains to which they are fused (19). Split-inteins have been successfully exploited to generate split proteins used in other expression systems (20, 21), and we sought to use them here to reconstitute wild-type Gal4 from two functionally inert fragments.

In a series of pilot experiments in S2 cells, we tested three different cysteine residues to split Gal4 into two nonfunctional fragments and four different split-intein systems (see *Materials and Methods* for full description) and ultimately identified the most potent system, which we refer to as “split-intein Gal4.” In this system, the Gal4 protein is split into two fragments: an N-terminal 20 amino acid portion (Gal4^N-int^) and the remaining C-terminal 861 amino acids (Gal4^C-int^), each flanked by components of the highly active *gp41-1* split-intein sequence (19) (Figs. 1 and 2*A*). When these two fragments are coexpressed in a cell, the split-intein activity is predicted to reconstitute the full wild-type Gal4 protein, which should be repressible by Gal80 (Figs. 1 and 2*A*). Previous studies in *Caenorhabditis elegans* have demonstrated a related approach, in which a DNA-binding domain and an AD are transspliced via *gp41-1* split-intein (20). However, in that approach, a VP64 AD is used instead of the native Gal4 domains, and thus this system is not repressible by Gal80.

#### NanoTag split-Gal4.

“NanoTags” are short epitope tags (<25 amino acids) that are recognized with very high affinity by single-domain nanobodies. Recently, two high-affinity NanoTags, 127D01 and VHH05, have been adapted for a variety of applications in vivo in *Drosophila* (22). We designed a split-Gal4 system based on the affinity of the 127D01 tag and its genetically encoded nanobody, Nb127D01. In a series of pilot experiments in S2R+ cells, we observed that Gal4DBD-Nb127D01 combined with Gal4AD-1x127D01 drove only very weak expression. However, when we fused three Nb12701 nanobodies in tandem to a Gal4DBD domain (Gal4DBD-3xNb127D01), making it capable of recruiting three Gal4-AD molecules to each Gal4DBD domain, we observed robust transgene expression. We refer to this combination as NanoTag split-Gal4.

### Both Split-Intein Gal4 and NanoTag Split-Gal4 Function in *Drosophila* Cell Culture.

To test the transcriptional activation strength of each system in cell culture, we transiently transfected either split-intein components (Gal4^N-int^ and Gal4^C-int^) or NanoTag Split-Gal4 components (Gal4DBD-3xNb12701 and Gal4AD-1x127D01), all driven by a constitutive *Actin5c* promoter, into S2R+ cells, along with a green fluorescent protein reporter (UAS:GFP). As positive controls, we transfected full-length Gal4 and standard split-Gal4 components, Zip-Gal4DBD and p65-Zip. Two days after transient transfection, we observed strong GFP expression for both split-intein Gal4 and NanoTag split-Gal4, at similar levels to Gal4 itself or to the existing split-Gal4 system (*SI Appendix*, Fig. S1).

We tested whether split-intein Gal4 and NanoTag Gal4 were repressible by Gal80 in S2R+ cells by cotransfecting these components with pTub:Gal80. As expected, wild-type Gal4, but not the existing split-Gal4 system (Gal4DBD-p65), was strongly repressed by Gal80 (*SI Appendix*, Fig. S1). Strikingly, both split-intein Gal4 and NanoTag split-Gal4 exhibited strong repression by Gal80, albeit slightly weaker than that observed for wild-type Gal4 (*SI Appendix*, Fig. S1). We conclude that both approaches offer robust transcriptional activation at levels similar to the existing split-Gal4 system, but have the critical advantage that they are also sensitive to the Gal80 repressor.

While both approaches showed promise in S2R+ cells, the 3:1 stoichiometry of Gal4AD:Gal4DBD in the NanoTag system suggested that this approach might require higher levels of Gal80 than the split-intein Gal4 approach to achieve the same level of repression. Since Gal80 expression levels in vivo will generally vary from cell type to cell type for any given Gal80 line and high sensitivity is therefore desirable, we chose to focus on the split-intein Gal4 system for additional in vivo testing.

### The Split-Intein Gal4 System Activates High Levels of Intersectional UAS-Driven Expression In Vivo.

In order to be a broadly useful tool in vivo, split-intein Gal4 must meet three criteria. It must: 1) *drive robust expression* in vivo, at levels similar to existing split-Gal4 or Gal4 lines; 2) *drive clean intersectional labeling* that is not “leaky,” and includes only those cells expressing both Gal4^N-int^ and Gal4^C-int^ components; 3) *be repressible* using existing Gal80^ts^ lines.

To characterize split-intein Gal4 in vivo, we used CRISPR-mediated knock-in transgenesis to insert split-intein components into various genes with well-characterized expression patterns. We first selected two genes expressed in specific cell types of the midgut: *Myo1A* (aka *Myo31DF*) which is expressed in enterocytes (ECs) (23), and *esg,* which is expressed in intestinal stem cells (ISCs) (24). To permit direct comparison with the current split-Gal4 system, we also generated knock-ins of ZipGal4DBD into the same positions within *esg* and *Myo1A*. To create these knock-ins, we adapted the “drop-in” cloning method (25) to generate homology-driven repair (HDR) donor plasmids that would insert an in-frame T2A sequence, followed by the split-intein Gal4 or split-Gal4 component, into an early exon of the target gene. We also generated ubiquitously expressed split-intein Gal4 components, driven by the *alphaTubulin48B* promoter, to use as “tester” lines to visualize the complete expression pattern of each knock-in.

We crossed *Myo1A-T2A-Gal4^N-int^* to the *tub-Gal4^C-int^; UAS:2xEGFP* (enhanced green fluorescent protein) tester line and observed cell type–specific expression in larval ECs, at levels statistically indistinguishable from the original split-Gal4 system (mean pixel intensity measured in *n =* 4 larval guts; t(6) = 0.9325, *P* = 0.3871) (Fig. 2*B*). Similarly, *esg-T2A-Gal4^N-int^* ∩ *tub-Gal4^C-int^* (hereafter we follow the convention of using the ∩ symbol to indicate intersectional labeling) drove specific expression in ISCs at similar levels to the standard split-Gal4 system (*n =* 3 larval guts; t(4) = 2.22; *P* = 0.091) (Fig. 2*B*). These results indicate that the split-intein Gal4 system functions robustly in vivo. Expression in the midgut was specific for the two targeted cell types, indicating that the Gal4^C-int^ fragment did not support leaky expression.

We then tested whether the split-intein Gal4 approach would successfully drive intersectional expression using two cell type–specific knock-in lines. As *esg* and *Myo1A* are not coexpressed in the gut, we knocked *Gal4^C-int^* into the *Delta* (*Dl*) gene, which is also expressed in ISCs (26). As expected, *esg* ∩ *Dl* expression was observed in adult ISCs (Fig. 2*C*). Importantly, the expression of *Dl* in the anterior hindgut was not observed in the *esg* ∩ *Dl* intersection, providing additional evidence that the split-intein system is not leaky (Fig. 2*C*).

Thus, the split-intein Gal4 system satisfies the first two criteria identified above: it drives expression at similar levels to the existing split-Gal4 system, and expression can only be detected in cells coexpressing both components.

### Split-Intein Gal4 Is Repressible by Gal80^ts^.

**Fig. 3.**
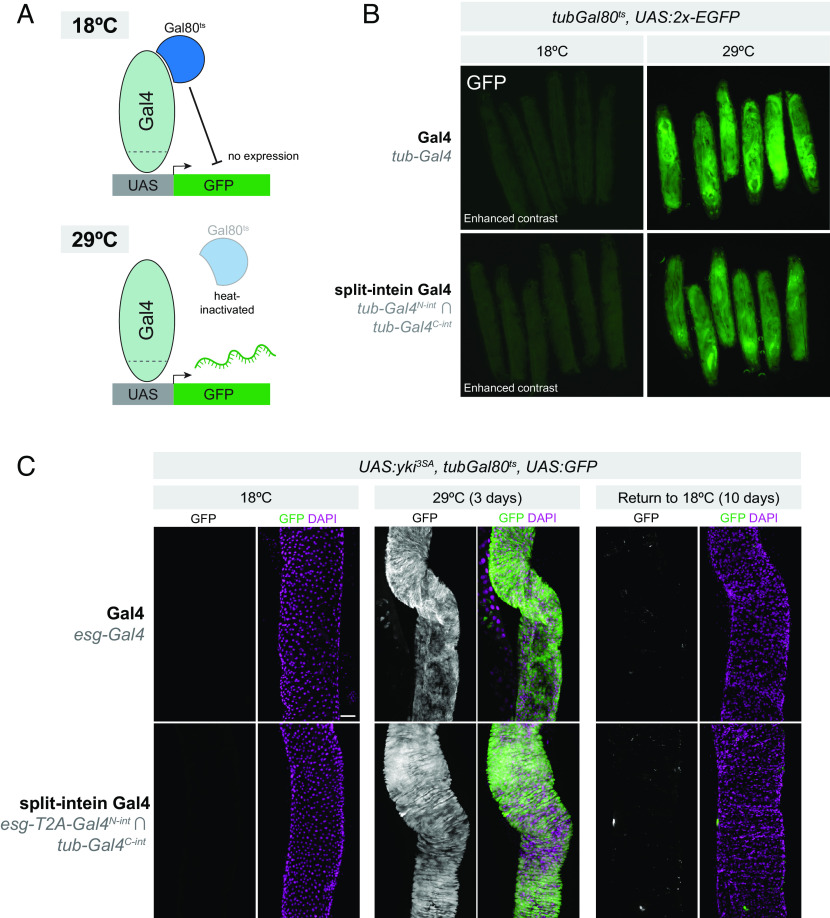
Split-intein Gal4 is repressible by Gal80. (*A*) Cartoon diagram of temperature-inducible expression using Gal80^ts^. (*B*) *tub-Gal4^N-int^* ∩ *tub-Gal4^C-int^ tub-Gal80^ts^* > *UAS:2xEGFP* expression is repressed at 18 °C and highly active at 29 °C, as is *tub-*Gal4, *tub-*Gal80^ts^ > UAS:2xEGFP. Note that the 18 °C images are shown with increased gain relative to the 29 °C images in order to visualize the presence of larva. (*C*) Recapitulation of a well-characterized adult stem cell tumor system using split-intein Gal4. *esg-Gal4^N-int^ ∩ tub-Gal4^C-int^, tub-*Gal80^ts^ > UAS:*yki^3SA^* in adult ISCs can be repressed throughout development and adult stages by *tubGal80^ts^* and reversibly activated using temperature shift to 29 °C. Anterior is up, scale bar in (*C*) is 50 µm.

The split-intein Gal4 system should seamlessly reconstitute wild-type Gal4, which, unlike the original split-Gal4 system, is repressible by Gal80 (Fig. 3*A*). To confirm this is the case, we generated larvae expressing both *tub-*Gal4^N-int^ and *tub-*Gal4^C-int^ as well as *tub-Gal80^ts^* and a *UAS:2x-EGFP* reporter.

When *tub-Gal4^N-int^* ∩ *tub-Gal4^C-int^, tub-Gal80ts* > *UAS:2xEGFP* larva were grown at 18 °C, no EGFP could be detected, similar to standard *tub-Gal4, tub-Gal80^ts^ > UAS:2xEGFP* larvae (Fig. 3 *B*, *Left*). However, when grown at 29 °C, strong EGFP expression was observed (Fig. 2 *B*, *Right*). We also confirmed that, in the absence of Gal80^ts^, the split-intein Gal4 system does indeed drive EGFP expression at 18 °C (*SI Appendix*, Fig. S2), indicating that the lack of EGFP expression at 18 °C is not the result of compromised split-intein trans-splicing, and is indeed due to Gal80 repression. These results also demonstrated that, like wild-type Gal4, split-intein Gal4 activity increases with temperature (27) (*SI Appendix*, Fig. S2). These results show that split-intein Gal4 is repressible by Gal80.

To confirm that the Gal80 repression of split-intein Gal4 is sufficiently potent to fully repress strong, dominant phenotypes at 18°C, we turned to a widely used tumor model in the adult gut. When activated *yki* is expressed in ISCs using *esg*-Gal4, it generates severe tumor phenotypes in the adult gut (28–30). We used the split-intein Gal4 system to drive activated *yki* (*UAS:yki^3SA^*) in adult ISCs (*esg-Gal4*^*N-int*^ ∩ *tub-Gal4*^*C-int*^), in the presence of *tub-Gal80^ts^.* Grown at 18 °C, these flies developed and eclosed normally, and displayed no EGFP expression or tumor growth in the ISCs, similar to the corresponding Gal4 system (Fig. 3 *C*, *Left*). However, after a 3-d temperature shift, we observed a dramatic tumor phenotype indistinguishable from those produced by *esg-Gal4* (Fig. 3 *C*, *Middle*). Further, we could re-repress this phenotype and the associated EGFP expression by shifting these flies back to 18 °C for 10 d (Fig. 3 *C*, *Right*). Altogether, these experiments confirm that split-intein Gal4 drives high levels of expression, is not leaky, and is repressible by existing Gal80^ts^ reagents.

The gene-specific split-intein Gal4 drivers described above were generated using CRISPR-based knock-ins, in order to fully recapitulate the endogenous expression pattern of the target gene. However, many researchers may wish to create Gal80-sensitive split-intein Gal4 drivers using specific enhancer fragments, as has been done successfully for thousands of split-Gal4 lines that have been generated for the VT split-Gal4 collection (6, 7). To facilitate this approach, we modified the pBPZpGAL4DBD and pBPp65ADZp destination vectors (12) to encode split-intein Gal4 components. These vectors are compatible with the well-established Gateway LR–based cloning workflow for generating enhancer fragment–driven split-Gal4 vectors (12, 31). To demonstrate the effectiveness of this approach, we selected a genomic fragment known to drive expression in the adult gut ISCs, VT024642 (13), and cloned this fragment into our pBP-Gal4^N-int^ destination vector. As predicted, *VT024642-Gal4^N-int^* ∩ *tub-Gal4^C-int^* drove strong, specific expression in adult ISCs (*SI Appendix*, Fig. S3.)

### Split-Intein Gal4 Components Can Be Adapted to GeneSwitch for Drug-Inducible Intersectional Labeling.

Having established that split-intein Gal4 is highly effective, we reasoned that this system should also be adaptable to the drug-inducible GeneSwitch system (32). In GeneSwitch, the Gal4DBD (the first 93 amino acids of the Gal4 protein) is fused to an RU486-sensitive ligand-binding domain (PR-LBD) and a p65 transcriptional AD. In the absence of RU486 (RU), the GeneSwitch complex is inactive, whereas in the presence of RU, the complex undergoes a conformational change that allows for the transcriptional activation of UAS-driven transgenes.

**Fig. 4.**
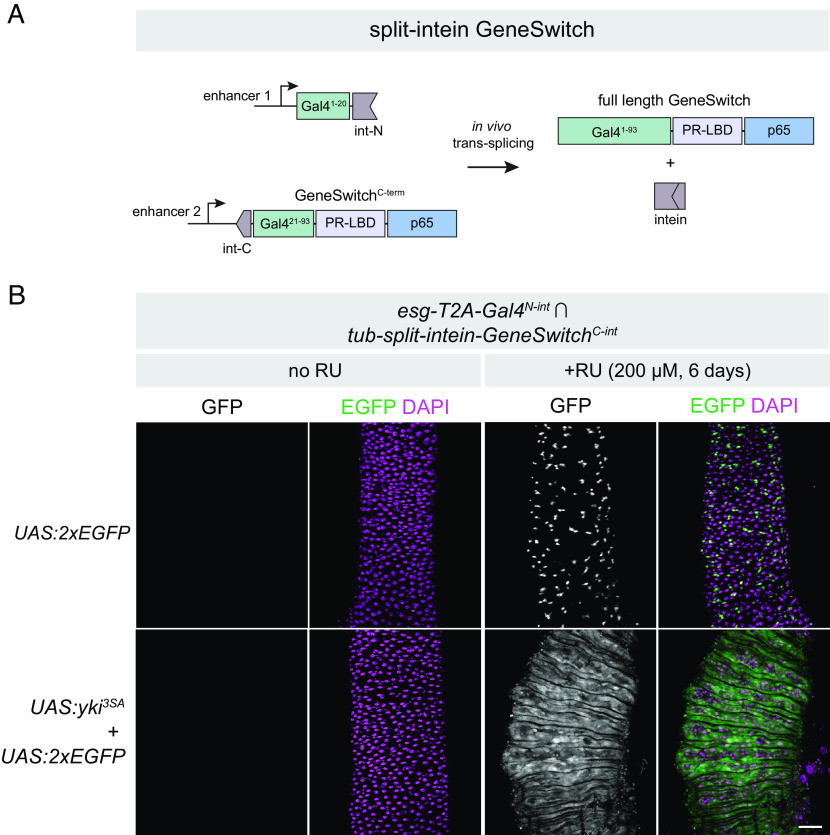
Drug-inducible intersectional labeling using split-intein GeneSwitch (*A*) Cartoon schematic of the split-intein GeneSwitch system, not drawn to scale. The same N-terminal fragment of Gal4 used in split-intein Gal4 can be combined with the C terminus of the GeneSwitch system, which includes amino acids 21 to 93 of Gal4, a progesterone ligand-binding domain (PR-LBD), and the p65 transcriptional activator. (*B*) Drug-inducible ISC tumor model using the *esg*-Gal4^N-int^ *∩ tub-*GeneSwitch^C-int^ > UAS:*yki^3SA^*. Anterior is up. (Scale bar, 50 µm.)

We noted that the Gal4^N-int^ fragment, compromising the first 20 amino acids of Gal4, could be compatible with the corresponding C-terminal region of GeneSwitch (Gal4^21-93^-PR-LBD-p65 aka GeneSwitch^C-int^) flanked by a split-intein (Fig. 4*A*). In other words, the same Gal4^N-int^ lines could be crossed to either a Gal4^C-int^ for split-intein Gal4 expression, or to a GeneSwitch^C-int^ line for split-intein GeneSwitch expression. To test this, we generated a transgenic line expressing *split-intein-GeneSwitch*^*C-int*^ under the control of the *tub* promoter. We crossed *tub-split-intein-GeneSwitch^C-int^; UAS:2xEGFP* to *esg-T2A-Gal4^N-int^* and split the F1 adult flies into RU- and RU+ minus treatments for 6 d. In the absence of RU, we observed no GFP expression in the adult gut, whereas flies fed RU-containing food displayed strong and specific GFP in adult ISCs (Fig. 4 *B*, *Top*). In parallel, we crossed *tub-split-intein-GeneSwitch^C-int^; UAS:2xEGFP* to *esg-T2A-Gal4^N-int^; UAS:yki^3SA^* to test whether we could successfully regulate tumor growth via RU feeding. While RU- flies displayed no EGFP expression and wild-type gut morphology, RU+ flies displayed strong ISC tumor phenotypes resembling those observed using either Gal4 or split-intein Gal4 (Fig. 4 *B*, *Bottom*). Thus, the split-intein GeneSwitch system successfully combines intersectional genetic labeling with the RU- inducibility of GeneSwitch.

Importantly, the GeneSwitch system has been shown to be leaky in some tissues, with detectable expression in the absence of RU (32–34). Given that split-intein GeneSwitch simply reconstitutes the existing GeneSwitch protein, we predicted this leaky expression would also be the case with split-intein GeneSwitch. We tested this by crossing the ubiquitously expressed *tub-split-intein-GeneSwitch^C-int^*tester line to three additional Gal4^N-int^ lines: *Myo1A-T2A-Gal4^N-int^*, *Dl-T2A-Gal4^N-int^*, and *tub-Gal4^N-int^*. While we observed clean RU-dependent expression in adult ISCs with both *esg* and *Dl* (*SI Appendix*, Fig. S4 *A* and *B*), we observed leaky expression in a portion of the adult gut using *Myo1A* (*SI Appendix*, Fig. S4*C*), as well in the larval gut when using the *tub* promoter (*SI Appendix*, Fig. S4*D*). Thus, as with existing GeneSwitch reagents, it will be important for researchers to carefully characterize the RU- and RU+ expression patterns for split-intein GeneSwitch lines.

### Mapping scRNA Clusters to Anatomy Using Split-Intein Gal4 Drivers.

One particularly promising use of intersectional labeling techniques such as split-intein Gal4 is to characterize the many transcriptionally defined “clusters” of cells that are identified using scRNAseq. Single-cell and single-nuclei transcriptomic atlases are now available for many individual *Drosophila* tissues, as well as for the entire adult body (35). These atlases identify many different distinct cell types within a given tissue based on transcriptional similarity, many of which remain uncharacterized either anatomically or functionally. In most cases, a single genetic marker is insufficiently specific to label a cluster, and a minimum of two coexpressed genes are generally required to demarcate a cluster (36). Thus, intersectional genetic labeling approaches are a promising tool to interrogate hypotheses generated via scRNAseq. The promise of this approach has recently been piloted in the *Drosophila* optic lobe (16).

**Fig. 5.**
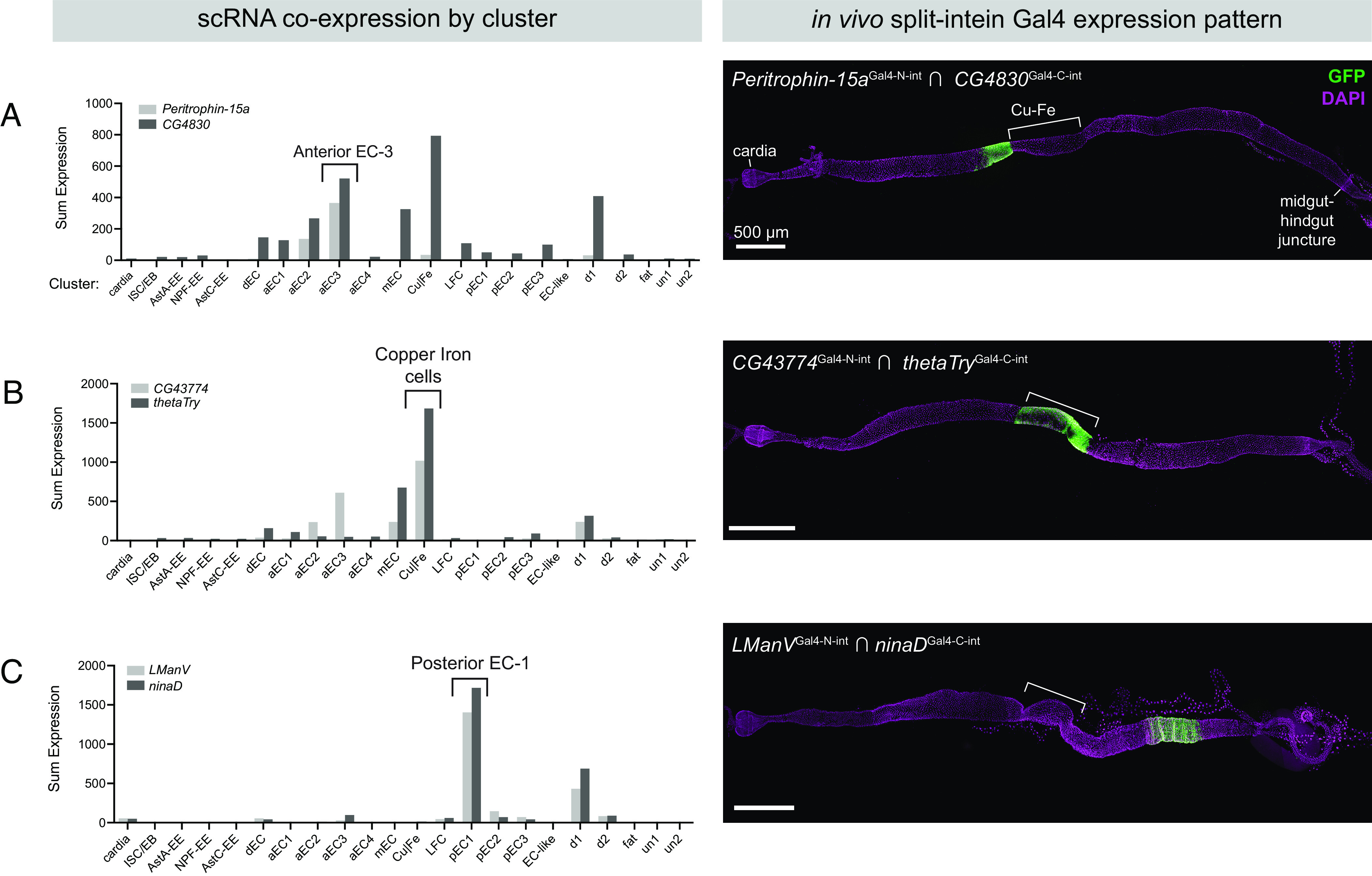
Mapping scRNA clusters to anatomy using split-intein Gal4 lines based on NS-Forest v2 predictions. Based on a gut-specific scRNA dataset from Hung et al. (37), the NS-Forest v2 algorithm identified pairs of genes to mark specific clusters in the adult gut. Left: Summed expression in each of the 22 clusters is shown on the *Left*. *Right*: In vivo expression of split-intein Gal4 lines in adult guts. Brackets indicate the approximate position of the copper and iron cell (Cu–Fe) region. (*A*) *Peritrophin-15a* ∩ *CG4830* drives expression in a band of enterocytes anterior to the copper cells. (*B*) *CG43774* ∩ *thetaTry* drives expression in the copper and iron cells. (*C*) *LManV* ∩ *ninaD* drives expression in a posterior band of enterocytes. Anterior is to the left.

To explore how the split-intein Gal4 system can leverage and inform scRNAseq datasets, we began with a recent atlas of the adult midgut, which identified 22 transcriptionally distinct cell types (37). To pick pairs of genes that uniquely mark scRNA clusters, we implemented a recently described gene selection algorithm, NS-Forest version 2.0 (36). NS-Forest v2 is a machine learning algorithm that estimates the minimum number of marker genes that can be used to uniquely define scRNAseq clusters. Using NS-Forest v2 to guide gene pair selection, we generated transgenic split-intein Gal4 lines to mark three of these clusters in vivo: 1) aEC-3, predicted to be a subset of anterior ECs, marked by *Peritrophin-15a* ∩ *CG4830;* 2) iron and copper cells, a functional analog of the human stomach located midway between the anterior and posterior of the midgut, marked by *CG43774* ∩ *thetaTry*; and 3) pEC-1, predicted to be a subset of posterior ECs, marked by *LManV* ∩ *ninaD* (Fig. 5).

We generated split-intein Gal4 lines for each of these three pairs and examined their expression using UAS:2xEGFP. In each case, the expression pattern conformed well with the predicted location of the cell cluster. Our aEC3 split-intein Gal4 line *Peritrophin-15a* ∩ *CG4830* drove expression in a band of ECs anterior to the copper cells, corresponding to the “A3” region identified by (38) (Fig. 5*A*). This band of expression had a sharp posterior border, but we also observed patchy expression extending anteriorly from the aEC3 cluster (Fig. 5*A*). This may reflect the in silico prediction of lower levels of coexpression in the aEC2 cluster, which is transcriptionally very similar to aEC3 (38) (Fig. 5*A*). The split-intein Gal4 line *CG43774* ∩ *thetaTry,* predicted to express in iron and copper cells, drove expression in precisely this region (Fig. 5*B*), and the pEC-1 line *LManV* ∩ *ninaD* drove expression in a band of ECs posterior to the copper cells (Fig. 5*C*). Thus, using in silico predictions to guide gene selection, we were able to successfully use split-intein Gal4 to label anatomically distinct populations of cells along the anterior–posterior axis of the adult midgut, demonstrating the potential power to identify and functionally characterize cell types identified via scRNAseq studies.

### Developing an Algorithm to Pick Cluster-Specific Gene Pairs with Minimal Coexpression across the “Whole-Body” scRNA Dataset.

Subsequent to the publication of the midgut-specific scRNAseq dataset described by (37) and utilized above, the Fly Cell Atlas Consortium published scRNAseq datasets covering many additional adult *Drosophila* tissues, as well as a whole-body dataset (35). As the set of potential off-target clusters now spans the whole body, it becomes more difficult to obtain gene pairs that mark a specific cluster. In particular, for each of the midgut-relevant gene pairs predicted by NS-Forest v2, we examined the in silico coexpression of these gene pairs across the entire body of Fly Cell Atlas dataset. For the large majority of gene pairs, we observed that the NS-Forest v2 gene pairs had a high degree of coexpression in multiple other tissues. This has practical implications: it is crucial to identify intersectional genetic drivers that are exclusively expressed in specific tissues, with no additional expression anywhere else in the body.

We therefore sought to develop an algorithm that would specifically identify cluster-specific gene pairs that maximize coexpression in the cluster of interest, while minimizing additional coexpression in both the tissue of interest, as well as across other scRNAseq datasets from the same organism. To do so, we developed a gene-selection algorithm that we call “Two Against Background” or TAB.

**Fig. 6.**
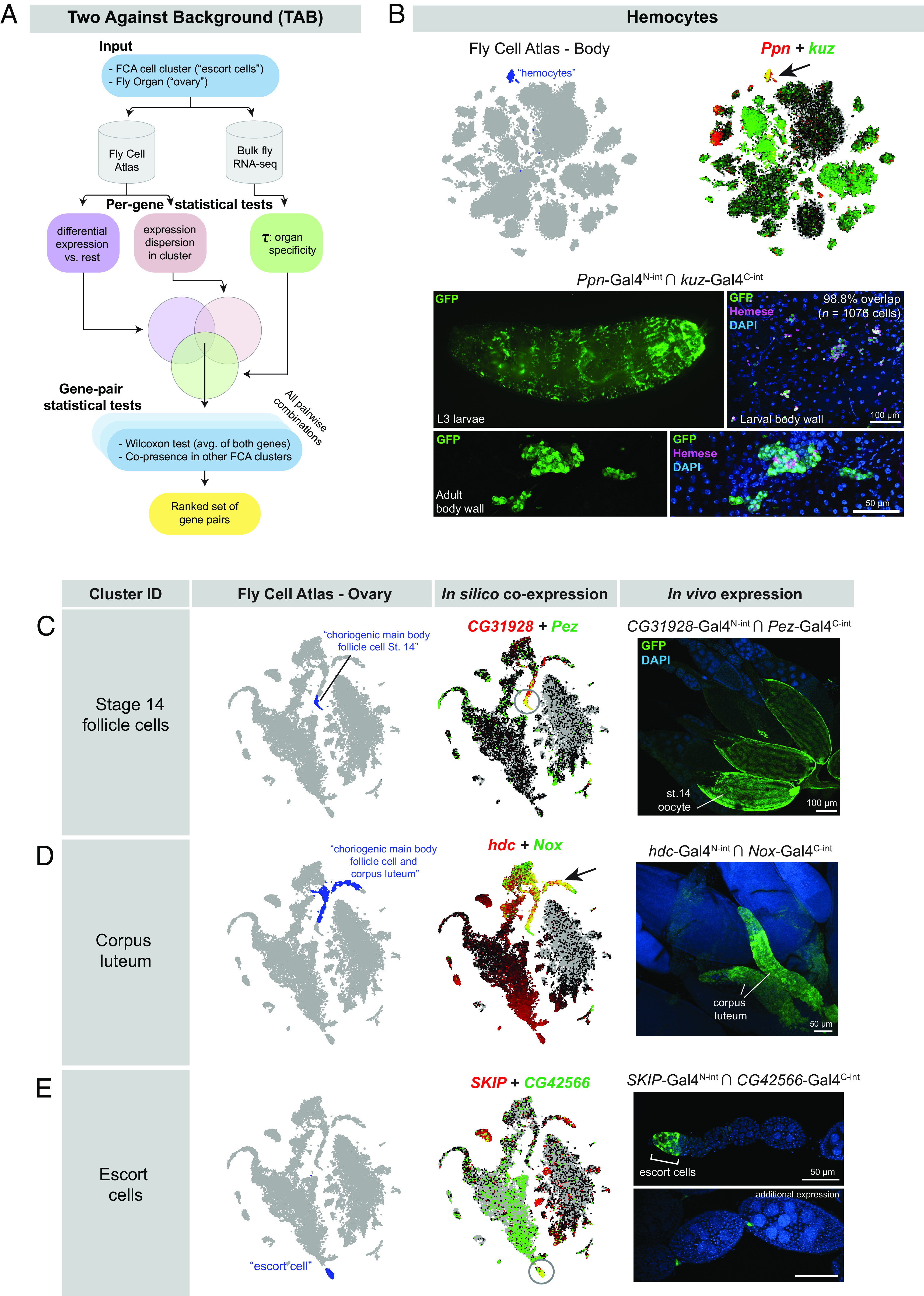
Characterization of split-intein Gal4 lines based on Two Against Background (TAB) algorithm predictions. (*A*) Schematic of the TAB algorithm to pick gene pairs that specifically mark scRNAseq clusters. (*B*) Hemocyte-specific split-intein Gal4 line based on TAB gene pairs. *Top* *Left*: Fly Cell Atlas 10× “Body” atlas, with hemocyte cluster indicated in blue. *Top* *Right*: In silico prediction of *Ppn* and *kuz* coexpression in the hemocyte cluster, with coexpression shown in yellow. *Bottom*: In vivo expression of the *Ppn* ∩ *kuz* split-intein Gal4 driver in larval and adult hemocytes. Hemocytes are costained with the H2 antibody against Hemese, a larval pan-hemocyte marker. (*C*–*E*) In silico predictions of coexpression in three different clusters from the FCA “Ovary” dataset and in vivo coexpression in the indicated cell types of the ovary. scRNAseq data are screenshots from the FCA data viewer for the “10×, stringent” datasets.

The TAB algorithm is schematized in Fig. 6*A*. Briefly, TAB incorporates three features to guide gene pair selection: First, it incorporates bulk RNAseq data, when available, to supplement scRNAseq expression estimates. Second, it emphasizes selecting genes with robust within-cluster expression profiles that are stable and not highly variable. Third, to calibrate the importance of cluster specificity relative to these robustness considerations, it employs a hyperparameter optimization approach by incorporating rankings by a researcher who is blinded to the parameters.

As input, TAB requires both the cell cluster (e.g., “escort cells”) and the containing tissue (e.g., “ovary”). To ensure that the genes being selected are well expressed in the tissue, we crossreference the gene’s bulk scRNAseq expression levels in the corresponding organ. While the organ-level resolution is coarser than scRNAseq cell clusters, the higher quality of bulk expression data is a valuable corrective for noisy scRNAseq expression estimates. To quantify the specificity of any gene to the organ of interest, we use the Tau statistic. In addition, we require that the gene’s within-cluster expression be stable and not highly variable, i.e., the dispersion (variance/mean) of the gene expression in the cluster is limited.

In the TAB algorithm, a candidate set of gene pairs is created for each cluster of interest using the intersection of three metrics: the Tau statistic, dispersion metrics, and *t* test of differentiation against all other clusters (Fig. 6*A*). From this candidate set, we evaluate all pairwise combinations of genes and select pairs that are effective at distinguishing the cluster of interest from others. One of the metrics we consider is the number of other clusters where both the candidate genes are potential markers. We also introduce an additional metric: we construct a metagene as the average of the two genes and perform the Wilcoxon rank-sum test to assess differential expression of the metagene in this cluster against other clusters. To optimize hyperparameters, a subset of gene pair predictions was analyzed by a researcher who was blinded to the parameters and who used the FCA data visualizer to rank the specificity of each gene pair in the cluster of interest, and across multiple FCA datasets. The final score for the candidate gene pair is a weighted combination of these metrics, and we output a ranked list of choices from which we select final gene pairs. Our implementation of the TAB algorithm is publicly available at https://github.com/rs239/tab_gene_markers.

To test the efficacy of the TAB algorithm, we used it to identify a “hemocyte” cluster from the FCA 10X “Body” dataset that uniquely coexpressed the genes *Ppn* and *kuz* (Fig. 6*B*). We then generated a pair of split-intein Gal4 lines designed to target the intersection of *Ppn* and *kuz* (Fig. 6*B*.) We observed specific expression of *Ppn* ∩ *kuz* in larval and adult hemocytes, verified by staining with the pan-hemocyte H2 antibody, which recognizes Hemese (Fig. 6*B*) (39). In larvae, 98.8% of Hemese+ cells were also GFP+ (*n* = 1,076 cells from two larvae), and we did not observe GFP+ cells that were Hemese negative. In adults, the H2 antibody did not appear to stain all of the morphologically identifiable hemocytes in the adult, consistent with previous observations (39). To test whether *Ppn* ∩ *kuz* drives expression in additional cell types, we examined expression in sagittally sectioned, decapitated adult flies, as well as in adult brains. In addition to specific expression in circulating hemocytes, we observed strong expression in a band of epithelial cells within the cardia, also known as the proventriculus, a structure at the foregut–midgut juncture (*SI Appendix*, Fig. S5*B*) (40). Interestingly, several previous studies have identified hemocyte-like cells at this location in the larva, which express independent hemocyte markers and may play an immune function (41, 42). We confirmed that the pan-hemocyte marker *hml*-*Gal4* is expressed in a subset of the cells at this anatomical position (*SI Appendix*, Fig. S5*B*). Thus, our split-intein Gal4 *Ppn* ∩ *kuz* line appears to be concordant with other hemocyte-specific markers and was not detected in other tissues.

We next generated a series of split-intein Gal4 lines to mark specific clusters based on the tissue-specific FCA “Ovary” atlas while minimizing expression in any other cluster at the level of the whole body. We selected three clusters from the FCA ovary dataset (Fig. 6 *C*–*E*) and examined the expression of the resulting split-intein Gal4 lines in vivo. In all the three cases, we observed the predicted expression. *CG31928* ∩ *Pez* predicted to express in follicle cells of stage 14 oocytes and drove GFP expression specifically in these cells (Fig. 6*C*). *hdc* ∩ *Nox* drove expression in the corpus luteum, a tissue composed of the follicle cells left behind after an egg is laid (43) (Fig. 6*D*). *SKIP* ∩ *CG42566* drove the predicted expression in escort cells at the anterior tip of each ovariole, although we also observed expression in stalk cells between each germline cyst (Fig. 6*E*).

To test whether these split-intein Gal4 lines drive expression in additional, nontargeted tissues, we examined expression in adult flies that had been sagitally sectioned, as well as in adult brains. Neither *CG31928* ∩ *Pez* nor *hdc* ∩ *Nox* drove detectable EGFP expression in any other adult tissue outside the desired cell type (*SI Appendix*, Fig. S5 *C* and *D*). *SKIP* ∩ *CG42566* drove expression in a small number of neurons in the brain (*SI Appendix*, Fig. S5*E*), but was otherwise undetectable outside of the germaria, in which escort cells reside. Thus, these lines were highly specific to the targeted cell type. These pilot experiments demonstrate that the TAB algorithm will be a useful tool to generate highly specific genetic tools for clusters identified by scRNAseq datasets, whether at the level of individual scRNAseq datasets, or across multiple datasets or the whole organism.

In addition to the NS-Forest v2 gene selection algorithm, a recent manuscript has presented an alternative gene selection algorithm for creating cell type–specific split-Gal4 lines in *Drosophila* based on scRNAseq datasets (16). Our TAB algorithm differs in several ways from the one described by Chen et al. TAB considers bulk tissue RNAseq datasets to enhance confidence in tissue-specific expression, and it also considers each member of the gene pair as equally important rather than implementing a greedy search based on a prespecified first gene. In addition, TAB uses well-established metrics of differential expression and gene specificity to create tests, rather than assuming a unimodal vs. bimodal gene expression model. Future in vivo experiments will be useful to empirically compare the relative utility of these and other approaches.

### One-Step Generation of Double-Knock-In Split-Intein Gal4 Lines Using Dual Drug Selection.

One technical bottleneck in the production of split-intein Gal4 lines or split-Gal4 lines is the fact that two independent transgenic lines must be created for each desired genetic driver. We reasoned that it may be possible to generate split-intein Gal4 lines in a single step by simultaneously generating double knock-in lines. To test this approach, we adapted our knock-in vectors to contain drug selection markers that were recently characterized as transgenesis markers in *Drosophila* (44). In this approach, each knock-in is marked by a separate drug resistance gene, and double knock-in transformants are selected by rearing larvae on food containing both of the relevant drugs.

**Fig. 7.**
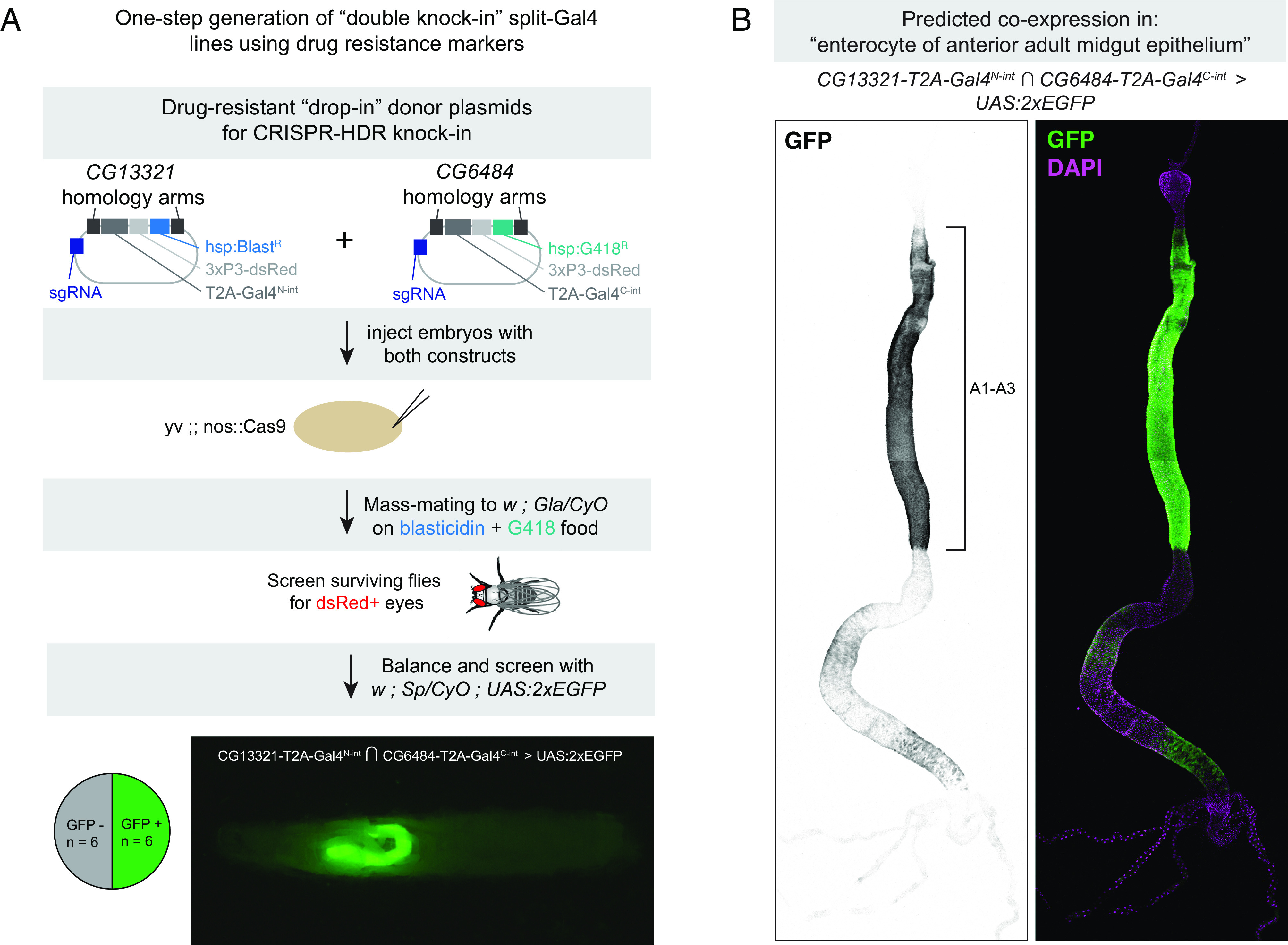
One-step generation of double-knock-in split-intein Gal4 line using drug selection markers. (*A*) Knock-in donor vectors containing drug resistance markers (Blast^R^ or G418^R^) are coinjected into embryos expressing germline-restricted Cas9, and the offspring of these injected G0s are screened for double drug resistance. Of the surviving F1, dsRed+ flies are screened for the ability to drive UAS:EGFP expression. Pie chart indicates the proportion of dsRed+ flies that successfully drove EGFP in the predicted cells, and image shows an L3 larva expressing EGFP in a portion of the gut, anterior to the left. (*B*) Expression pattern of *CG6484*-Gal4^N-int^ ∩ *CG13321-*Gal4^C-int^ in the adult gut, with anterior enterocyte regions A1-A3 (38) indicated with a bracket. Anterior is up.

We created a modified version of our Gal4^N-int^ donor vector containing a resistance gene for blasticidin (Blast^R^), and a version of our Gal4^C-int^ donor vector containing a resistance gene for G418 (G418^R^), and retained the fluorescent 3xP3-dsRed marker in both vectors. We used TAB to identify two pairs of genes which mark clusters from the FCA Gut atlas: *CG13321* ∩ *CG6484* to label “enterocyte of anterior adult midgut epithelium,” and *CG14275 ∩ CG5404* to label “hindgut.” For each gene pair, we generated two separate drug resistance knock-in vectors, with one construct resistant to blasticidin and the other resistant to G418 (Fig. 7*A*).

We injected a 1:1 mixture of these two vectors into *nos-Cas9* embryos and mass-mated the resulting injected G0 flies to a balancer stock, on food containing both blasticidin and G418 (Fig. 7*A*). Of the flies that survived, we selected flies with *dsRed+* eyes and screened these by crossing to a UAS:2xEGFP reporter. For *CG14275 ∩ CG5404,* we only recovered a small number of flies after drug screening, zero of which were *dsRed+*. However, for *CG13321* ∩ *CG6484,* of the 12 *dsRed+* flies we screened, six (50%) drove GFP expression, indicating successful one-step creation of double knock-ins in *cis* on chromosome 2R. These six lines drove strong EGFP expression throughout the anterior region of the midgut, from the posterior limit of the cardia to the anterior limit of the copper cells, as well as weaker, spotty expression in portions of the posterior midgut (Fig. 7*B*). Independently, we created separate knock-ins for *CG13321-*Gal4^N-int^ and *CG6484*-Gal4^C-int^ using our standard HDR vectors and confirmed that these lines drove expression in an identical pattern.

We note that both of these genes are located on Chromosome 2R, indicating that it would be challenging to use standard recombination genetics to create a single chromosome containing both inserts, which further demonstrates the value of making a one-step double knock-in. However, we note an important caveat. Specifically, the double-drug selection protocol was not 100% effective, as some dsRed-negative flies were observed after the first round of mating. In addition, 50% of the dsRed+ flies did not drive EGFP expression, which could indicate either that the two knock-in events occurred in *trans* on homologous chromosomes and were thus not captured via our screening procedure, or that only a single knock-in occurred. Thus, while the double knock-in strategy can serve to quickly generate split-intein Gal4 lines, it will require additional troubleshooting to be a reliable and scalable approach.

## Discussion

In the original description of the split-Gal4 system, it was noted that the replacement of the native Gal4 AD with the VP16 activator represented a trade-off: VP16 drove much stronger expression, but rendered split-Gal4 insensitive to repression by Gal80 (8). Here, we present an alternative split-Gal4 system that obviates the need for this trade-off by generating full-length wild-type Gal4 protein from two nonfunctional fragments, using self-splicing split-inteins. The split-intein Gal4 system combines the exquisite cell type specificity of split-Gal4 with the ability to temporally control Gal4 activity using existing Gal80^ts^ reagents. This system drives clean and specific transgene expression at similar levels to the existing split-Gal4 and Gal4 systems and is repressible by Gal80^ts^. Similar to Gal4 lines, additional spatial restriction of split-intein Gal4 activity should be possible using existing Gal80 lines. We believe that these advantages will make the split-intein Gal4 system a valuable addition to the toolkit available to the *Drosophila* research community for targeted transgene expression in specific cell types.

Targeting of specific cell types should be further facilitated by the widespread availability of scRNAseq datasets. As demonstrated here, such datasets can be leveraged to create intersectional split-intein Gal4 tools based on the knowledge of cell type–specific gene expression. This approach should allow researchers to test hypotheses generated by scRNAseq atlases and to de-orphan clusters of unknown anatomy or function. It will also aid in the creation of highly specific drivers for nearly all cell types and tissues in the fly and permit functional manipulations of these cell types with temporal control. To aid in the design of cell type–specific drivers, we have developed the TAB algorithm, which we believe will reduce the potential for coexpression outside a specific cluster of interest. Future characterization of the TAB algorithm across a variety of scRNAseq datasets will help further refine the cell type–specific tools available to the *Drosophila* research community.

To facilitate the creation of split-intein Gal4 lines, we have generated a plasmid tool kit to create split-intein Gal4 lines, either enhancer driven or via knock-in. For knock-ins, we provide plasmids for cloning via “long” homology arms (~1,000 bp), or via “drop-in cloning” using 200 bp fragments, which is what we use in this manuscript. These plasmids are diagrammed in *SI Appendix*, Fig. S6 and have been deposited in Addgene.

## Materials and Methods

Full methods are available in *SI Appendix*.

### Experimental Animals.

*Drosophila melanogaster* stocks were maintained and crossed on standard laboratory cornmeal food, and experiments were conducted at 18 °C, 25 °C, or 29 °C as indicated in the text. All adult experiments were performed in females. The new transgenic lines created in this study are described in *SI Appendix*, Table S1, and all genotypes are provided in *SI Appendix*, Table S2.

### Optimization and Cloning of Split-Intein Gal4 and NanoTag Split-Gal4 Components.

Design and cloning of components for both cell culture and in vivo, including “drop-in” cloning of knock-in vectors as well as promoter-driven constructs, is described in detail in Supplemental Index Materials and Methods.

### Testing Split-Intein Gal4 and NanoTag Split-Gal4 in S2R+ Cells.

*Drosophila* S2R+ cells (DGRC, 150) were cultured at 25 °C, in Schneider’s media (Thermo Fisher Scientific, 21720–024) with 10% fetal bovine serum (Sigma, A3912) and 50 U/mL penicillin-streptomycin (Thermo Fisher Scientific, 15070–063). S2R+ cells were transfected using Effectene (Qiagen, 301427) following the manufacturer’s instructions. Two hundred nanograms of plasmid DNA per well was transfected in 24-well plates. The cultured cells were imaged live 2 d after transfection on an InCell Analyzer 6,000 automated confocal fluorescence microscope (GE Healthcare Lifesciences).

### RU486 Treatment.

RU486 (Cayman Chemical Company Cat. No. 10006317) was added to standard fly food at a final concentration of 200 µm. For larval experiments, eggs were laid directly onto RU-containing food. For adult gut experiments, eggs were laid on and developed on standard food, and adults were transferred to RU-containing food for the indicated time.

### Drug Selection for Double Knock-Ins.

G418 (final concentration = 250 µg/mL) and blasticidin (final concentration = 45 µg/mL) were added to 25 mL of standard food in bottles and allowed to dry, uncovered, overnight in a fume-hood. Injected flies were mass-mated to balancer lines on drug food and flipped approximately every 3 d onto new drug-containing food. The surviving F1 offspring were screened for dsRed+ eyes, and any flies with dsRed+ eyes were then crossed to *w; Sp/CyO; 2xEGFP* to simultaneously balance and screen for double knock-ins of split-intein Gal4 components.

### Antibody Staining and Imaging.

For sagittal sections of whole flies, decapitated adult female flies were fixed overnight in 4% paraformaldehyde, then manually sectioned using a fine razorblade (Personna by AccuTec, Cat No. 74-0002). After antibody staining, bisected flies were placed in a drop of VECTASHIELD mounting media in a 35-mm, glass-bottom imaging µ-Dish (Ibidi, Cat. No. 81158). Tissues were dissected in PBS, fixed for 20 to 30 min in 4% paraformaldehyde, and stained using standard protocols. GFP was detected using either Alexa488-coupled anti-GFP (Invitrogen A21311, used at 1:400) or chicken anti-GFP (Aves Lab GFP1020, used at 1:2,000). Hemocytes were stained using the pan-hemocyte H2 antibody (39) (Gift of Andó lab, used at 1:100). Primary antibodies were detected with Alexa-488 or Alexa-555 coupled secondary antibodies (Molecular Probes). Confocal imaging was performed on either a Zeiss LSM 780 or Zeiss Axio Observer Z1 with a LSM980 Scan Head, with the “Tile Scan” feature for whole guts using system defaults. Whole-larva imaging was performed on a Zeiss AxioZoom microscope. Mean pixel intensity was measured using FIJI/ImageJ, based on maximum intensity projections, with GFP+ pixels selected as regions of interest.

### TAB Algorithm.

The scripts for TAB implementation are available at https://github.com/rs239/tab_gene_markers, and the algorithm is described in detail in Supplemental Index.

## Supplementary Material

Appendix 01 (PDF)

## Data Availability

All study data are included in the article and/or *SI Appendix*.
