# Supplementary material for: split-intein Gal4 provides intersectional genetic labeling that is repressible by Gal80
Source: Proc Natl Acad Sci U S A. 2023 Jun 5;120(24):e2304730120. doi: 10.1073/pnas.2304730120 (PMC10268248; doi:10.1073/pnas.2304730120)
Supplement: Supplementary file 1 — Appendix 01 (PDF) [file pnas.2304730120.sapp.pdf]

PNAS

Supporting Information for
split-intein Gal4 provides intersectional genetic labeling that is repressible by Gal80

Ben Ewen-Campen, Haojiang Luan, Jun Xu, Rohit Singh, Neha Joshi, Tanuj Thakkar, Bonnie Berger, Benjamin H. White, Norbert Perrimon

Corresponding Author: Norbert Perrimon
Email: perrimon@genetics.med.harvard.edu

This PDF file includes:

Full Methods
Figures S1 to S6
Tables S1 and S2
SI References

Supporting Information Text

Methods and Materials

Experimental animals. *Drosophila melanogaster* stocks were maintained and crossed on standard laboratory cornmeal food, and experiments were conducted at 18ºC, 25ºC, or 29ºC as indicated in the text. All adult experiments were performed in females. The new transgenic lines created in this study are described in **Table S1**, and all genotypes are provided in the Table of Genotypes below**.** The following previously described stocks and alleles were used:

tub::Gal4DBD ; UAS:2xEGFP (BL60298) - "split-Gal4 tester line"
tub::VP16[AD], UAS:2xEGFP (BL60295) - "split-Gal4 tester line"
w ; Sp / CyO ; UAS-2XEGFP (BL60293)
w ;; UAS-2XEGFP, tubGal80^{ts} (recombined from BL60293 and BL7018)
esg-Gal4, UAS:GFP, tubGal80^{ts} (Perrimon lab stock)
yki^{3SA} (BL28817)
w ;; tub-Gal4 (Perrimon lab stock)
actin:GeneSwitch (Perrimon lab stock)
hml-Gal4 > UAS:2XEGFP (BL30140)

Optimization and cloning of split-intein Gal4 components. To generate the split-intein Gal4 constructs, we proceeded in two steps. We first identified a suitable site to split the Gal4 molecule into inert fragments, and we then tested a variety of split intein pairs for their ability to reconstitute strong Gal4 activity. To generate transcriptionally inactive Gal4 fragments we sought splice sites that would divide the Gal4 DNA binding domain. We focused on serine and cysteine residues since these typically promote split intein splicing efficiency when located in the +1 position on the C-terminal side of the splice junction. We selected three cysteine residues within the Gal4 DNA binding domain (C^{21}, C^{28} and C^{31}) as candidate splice junctions and for each created complementary split intein Gal4 constructs using the hybrid split intein Npu $DnaE_N$ and Ssp $DnaE_C$ (45). To test the splicing efficacy of the three resulting pairs of split intein Gal4 constructs, we transfected S2 cells with plasmids encoding each pair and monitored the expression of a UAS-GFP reporter as described in (8). Constructs that split the Gal4 molecule at C^{28} failed to yield any Gal4 activity, whereas constructs using C^{21} and C^{31} as splice junctions were equally efficacious. The C^{21} residue was thus used as the splice junction for all further constructs. Preliminary tests *in vivo* with Npu $DnaE_N$ and Ssp $DnaE_C$ hybrid generated only low levels of Gal4 activity *in vivo*, so we next tested split intein Gal4 constructs using additional split inteins, including gp41-1 (19) and Cfa (46). Split intein Gal4 constructs made with gp41-1 exhibited the highest Gal4 activity and were selected for use *in vivo*.

Because GeneSwitch shares the same DNA binding domain with Gal4 (i.e. amino acids 2-93), split intein GeneSwitch constructs were generated using as the splice junction the same C^{21} cysteine residue. The split intein Gal4 and split intein GeneSwitch constructs thus share the same N-terminal Gal4[1-20] component.

To create the Gal4[1-20]-gp41-1inteinN aka $Gal4^{N\text{-}int}$ (HJP-277) and inteinC-Gal4[21-881] aka $Gal4^{C\text{-}int}$ (HJP-287) plasmids that served as the basis for future cloning into plasmids for transgenesis, we generated synthetic oligonucleotide gBlocks (Integrated DNA Technologies, Inc., Coralville, Iowa) containing the gp41-1 and Gal4 fragments, and cloned these into expression vectors using Infusion cloning (In-Fusion HD Cloning Plus, Cat. 638911, Takara Bio USA.)

Design and cloning of NanoTag split-Gal4 components and other constructs for experiments in S2R+ cells. All constructs for S2R+ experiments were cloned into an actin-driven expression construct derived from pAW-H2B-mCherry-12701 vector described in (22). This backbone was digested with *XbaI* and *NheI* enzymes, and various PCR-amplified inserts were

then added via Gibson Assembly (New England BioLabs Cat. No. E2611.) To generate pAw-Gal4DBD-3x127D01, we first cloned Gal4DBD-1xNb1207 by amplifying the Gal4DBD insert from pCRISPaint-T2A-Gal4-3xP3-RFP (47) and Nb127D01 from pAW-Nb127D01-GFP (22). We subsequently used Gibson Assembly to insert two additional Nb127D01 fragments. To generate pAW-NLSGal4AD-127D01, pAW-H2B-mCherry-127D01 was double-digested as described above, and the NLS-Gal4AD domain from a plasmid originally based on pActL-Gal4AD (Addgene 15303) and the 127D01 tag was inserted as part of the primer. The inserts for the original split-Gal4 components (pAW-Zip$^-$-Gal4DBD and pAW-p65-Zip$^+$) were amplified from pBPZpGAL4DBDUw (Addgene 26233) and pBPp65ADZpUw (Addgene 26234), respectively. pCaSpeR-tub-Gal80 was used to test for Gal80 sensitivity in S2R+ cells.

Testing split-intein Gal4 and Nanotag split-Gal4 in S2R+ cells. *Drosophila* S2R+ cells (DGRC, 150) were cultured at 25°C, in Schneider's media (Thermo Fisher Scientific, 21720–024) with 10% fetal bovine serum (Sigma, A3912) and 50 U/ml penicillin-streptomycin (Thermo Fisher Scientific, 15070–063). S2R+ cells were transfected using Effectene (Qiagen, 301427) following the manufacturer's instructions. A total of 200 ng of plasmid DNA per well was transfected in 24-well plates. The cultured cells were imaged live two days after transfection on an InCell Analyzer 6000 automated confocal fluorescence microscope (GE Healthcare Lifesciences).

Cloning and transgenesis of pTub-driven constructs. Plasmids were constructed using Gibson assembly (New England BioLabs Cat. No. E2611), as follows. A 2,595bp fragment of the *Drosophila alphaTubulin48B* promoter, followed by a 35bp minimal promoter and Kozak sequence, was PCR-amplified from pCaSpeR-tub-Gal80 (F: AAGCTTGCACAGGTCCTGTTCG R: GTTGCGGCCGCGGATCTG). This promoter was cloned upstream of either the 327bp open reading frame of Gal4$^{N\text{-}int}$ or the 2,697bb open reading frame of Gal4$^{C\text{-}int}$, in a backbone containing an SV40 3'UTR, an attB sequence and a mini-white marker (backbone originally from Addgene 140620). These constructs were inserted into the attP40 on the second chromosome using standard integrase-mediated transgenesis method.

Cloning of T2A knock-in vectors via "drop-in" cloning. To generate in-frame knock-ins of split-intein Gal4 components, we followed a modification of the "drop-in" cloning technique (25). For targeting *esg, Dl,* and *Myo1A*, we designed 200bp left and right homology arms, flanking a cloning cassette containing two inverted BbsI sites. These were synthesized and cloned into the pUC57_Kan_gw_OK backbone (25) by GeneWiz (Azenta Life Sciences). Upon *BbsI* digestion, these plasmids generate directional overhangs that allow for ligation of any insert with compatible overhangs. This backbone also includes a self-targeting sgRNA that linearizes the insert. In order to create an in-frame insert, the last nucleotide of the left homology arm was always the first nucleotide of a codon triplet in the target gene. Additionally, the last nucleotide of the left homology arm was never a thymine, which would create a TAG stop codon in our donor plasmid.

For *esg, Myo1A,* and *Dl* donor constructs, we generated drop-in-compatible split-intein Gal4 inserts via PCR. To do so, we first used Gibson assembly to subclone T2A-Gal4$^{N\text{-}int}$ or T2A-Gal4$^{C\text{-}int}$ into the "universal T2A HDR donor" backbone from (47), which includes a hsp70 3'-UTR and a 3XP3-dsRed transgenesis marker. We then PCR-amplified this insert (T2A-Gal4$^{N \text{ or } C\text{-}int}$-hsp70-3'UTR-3xP3-dsRed) using primers that include BsmBI sites designed to produce overhangs compatible with digested pUC57-homology arm plasmids. Following *BsmBI* digestion, these PCR fragments were ligated into BbsI-digested pUC57 donor constructs to create inserts flanked by 200bp homology arms. We also note that the Universal T2A HDR donor-Gal4$^{N\text{-}int}$ and -Gal4$^{C\text{-}int}$ are compatible with cloning ~1000bp homology arms as described in (47). sgRNAs were cloned into the pCFD3 vector (48), CRISPR knock-in transgenesis was performed as previously described (49).

For knock-ins generated based on scRNAseq clusters, we streamlined the drop-in cloning process as follows. We cloned the entire T2A-Gal4$^{N\text{-}int}$ and T2A-Gal4$^{C\text{-}int}$ inserts, including the *hsp70-3UTR* and *3xP3-dsRed*, each flanked by inverted BsmBI sites, into the pCRBluntII-TOPO backbone, so the inserts can be released via simple digestion rather than PCR. We also switched

to an updated pUC57 backbone, pUC57_Kan_gw_OK2 (50), which includes the genome-targeting sgRNA in the same backbone as the donor, so it does not need to be co-injected as an independent construct. The sgRNAs used to create these knock-in constructs are described in **Table S1**.

Drug-resistant knock-in donors were cloned using Gibson Assembly. We amplified the *hsp-BlastR* from Addgene 165911 and *hsp-G418R* from Addgene 165902 (44), with compatible overhangs to clone them downstream of the 3xP3-dsRed marker in the drop-in compatible split-intein Gal4 constructs described above.

Generation of plasmids for enhancer-driven split-intein Gal4. To create pBP-Gal4$^{N\text{-}int}$ and pBP-Gal4$^{C\text{-}int}$ for LR Gateway cloning of enhancer fragments, we used Gibson Assembly to replace the Gal4DBD sequence in pBPZpGAL4DBDUw (Addgene 26233). The 2.3kb enhancer fragment VT024642 was amplified from genomic DNA using these primers: F: cctcttcaacacgcccaccaaactg, R: ctgcggctgaccacatcgagaa, with "CACC" appended to the 5' end of the F primer to facilitate directional cloning into dTOPO-pENTR. LR Gateway cloning was conducted using standard protocols to generate VT024642-Gal4$^{N\text{-}int}$, and standard phiC31 transgenesis was used to integrate this construct into the attP40 site.

RU486 treatment. RU486 (Cayman Chemical Company Cat. No. 10006317) was added to standard fly food at a final concentration of 200 μm. For larval experiments, eggs were laid directly onto RU-containing food. For adult gut experiments, eggs were laid on and developed on standard food, and adults were transferred to RU-containing food for the indicated time.

Drug selection for double knock-ins. G418 (final concentration = 250 μg/mL) and blasticidin (final concentration = 45 μg/mL) were added to 25 mL of standard food in bottles and allowed to dry, uncovered, overnight in a fume-hood. Injected flies were mass-mated to balancer lines on drug food, and flipped approximately every 3 days onto new drug-containing food. The surviving F1 offspring were screened for dsRed+ eyes, and any flies with dsRed+ eyes were then crossed to *w ; Sp/CyO ; 2xEGFP* to simultaneously balance and screen for double knock-ins of split intein-Gal4 components.

Antibody staining and imaging. For sagittal sections of whole flies, decapitated adult female flies were fixed overnight in 4% paraformaldehyde, then manually sectioned using a fine razorblade (Personna by Accutec, Cat No. 74-0002). After antibody staining, bisected flies were placed in a drop of Vectashield mounting media in a 35mm, glass-bottom imaging μ-Dish (Ibidi, Cat. No. 81158.) Tissues were dissected in PBS, fixed for 20-30 minutes in 4% paraformaldehyde, and stained using standard protocols. GFP was detected using either Alexa488-coupled anti-GFP (Invitrogen A21311, used at 1:400) or chicken anti-GFP (Aves Lab GFP1020, used at 1:2000.) Hemocytes were stained using the pan-hemocyte H2 antibody (39) (Gift of Andó lab, used at 1:100.) Primary antibodies were detected with Alexa-488 or Alexa-555 coupled secondary antibodies (Molecular Probes.) Confocal imaging was performed on either a Zeiss LSM 780 or Zeiss Axio Observer Z1 with a LSM980 Scan Head, with the "Tile Scan" feature for whole guts using system defaults. Whole-larva imaging was performed on a Zeiss AxioZoom microscope. Mean pixel intensity was measured using FIJI/ImageJ, based on maximum intensity projections, with GFP+ pixels selected as regions of interest.

Two Against Background ("TAB") Algorithm. The scripts for TAB implementation are available at https://github.com/rs239/tab_gene_markers. The selection of marker genes is a critical step in implementing the split-intein Gal4 protocol and poses unique challenges that are not addressed by existing marker-gene selection approaches. We began by articulating specific design goals for the selection algorithm: robustness to errors in scRNA-seq measurements; facilitating exploration by allowing users to choose gene pairs from a preferred set of markers; and achieving high accuracy against the whole-body background of the organism, including individual clusters in other organs. We emphasize cluster-level accuracy because high expression of the marker genes in even one off-target cluster can make *in vivo* results difficult to interpret. Existing marker-gene

selection approaches are not well-suited to address these specific design objectives. Approaches that simply compare marker genes against an overall background may be unable to rule out niche cell-clusters that are a small part of the overall set, thus making the selection of precisely two markers challenging. NS-Forest v2, which was created specifically to identify a minimal set of genes to uniquely identify cell types, compares a target cluster against a set of other clusters but does not limit to marker sets with two genes. In contrast, TAB requires the identification of exactly two markers.

Our TAB algorithm addresses our design goals using an integrative approach that incorporates bulk RNA-seq data, an emphasis on selecting genes with robust expression in the cluster, and hyperparameter optimization by single-blind expert evaluations. We require the input of both the cluster of interest, as well as the anatomical tissue where this cluster resides. To ensure that the genes being selected are sufficiently highly expressed in the tissue, we examine the gene's bulk scRNA-seq expression levels in the corresponding organ. The organ-level resolution is a valuable corrective for relatively noisy expression estimates from scRNA-seq atlases. We use the Tau statistic to quantify the specificity of any gene to the organ of interest. In addition, we minimize the dispersion (= variance/mean) that the gene's within-cluster expression, to ensure that it is not highly variable within the cluster-of-interest.

We use an intersection of three metrics to generate a list of candidate gene pairs: the Tau and dispersion metrics, along with t-test of differentiation against all other clusters. From this candidate set, we evaluate all pairwise combinations of genes and select pairs that are effective at distinguishing the cluster of interest from others. As in NS-Forest v2, one of the metrics we consider is the number of other clusters where both the candidate genes are potential markers. We also construct a metagene as the average of the two genes, and perform the Wilcoxon rank-sum test to assess differential expression of the metagene in this cluster against other clusters. TAB outputs a ranked list of gene pairs, based on a weighted combination of these metrics.

The TAB algorithm includes a key innovation of single-blind expert-guided hyperparameter optimization to address the challenges of fine-tuning the statistical tests and metrics. With many FCA clusters being novel and poorly characterized, it is difficult to obtain ground-truth, experimentally-supported annotations of cluster markers that could be leveraged for calibrating TAB. To address this issue, we adopted an expert-guided approach: one researcher selected a set of cell clusters for evaluation, while another performed the computations for a selection of hyperparameter choices. On a subset of the clusters, the gene-pairs predictions were stripped off their associated hyperparameters and provided to the first researcher to analyze, based on inspection of the FCA data portal. The expert scores for the gene pairs were used to refine the hyperparameters, and the computational analysis was re-run on a different subset of the test cluster set. This iterative process was repeated until a satisfactory success rate was achieved. Through this single-blind expert-guided hyperparameter optimization, TAB is able to select optimal gene-pairs with high accuracy and reliability, even in the absence of ground-truth annotations.

To summarize, our TAB algorithm features several new features to the challenge of marker-gene selection. Firstly, it incorporates bulk RNA-seq data to supplement scRNA-seq expression estimates when available. Secondly, it emphasizes selecting genes with robust within-cluster expression profiles that are stable and not highly variable. Lastly, it employs an expert-guided approach for hyperparameter optimization, providing a principled and unbiased fine-tuning process.

Supplemental Figures

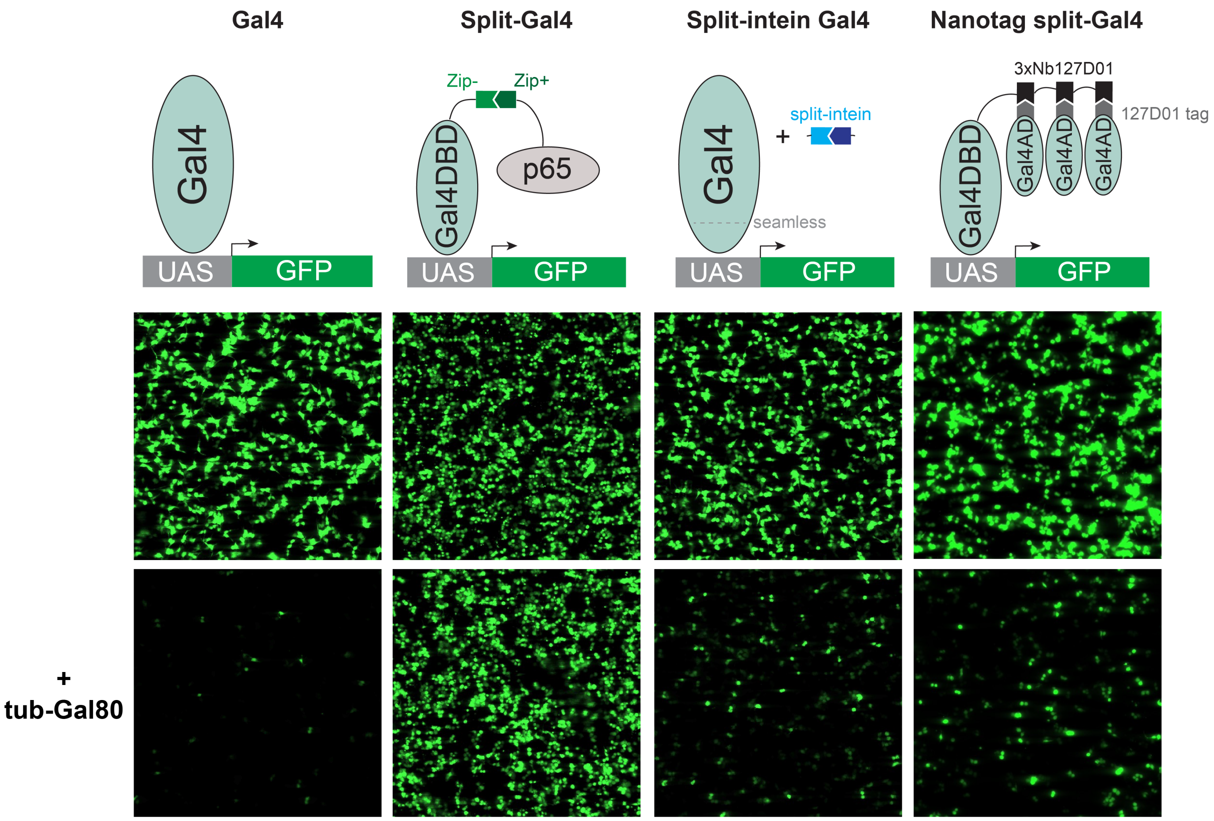

Fig. S1. Pilot characterization of split-intein Gal4 and Nanotag split-Gal4 in S2R+ cells. Plasmids encoding Gal4, split-Gal4, split-intein Gal4, or Nanotag split-Gal4, each driven by a constitutively expressed Actin promoter, were transiently transfected into S2R+ cells, either with or without co-transfection of the Gal80 repressor.

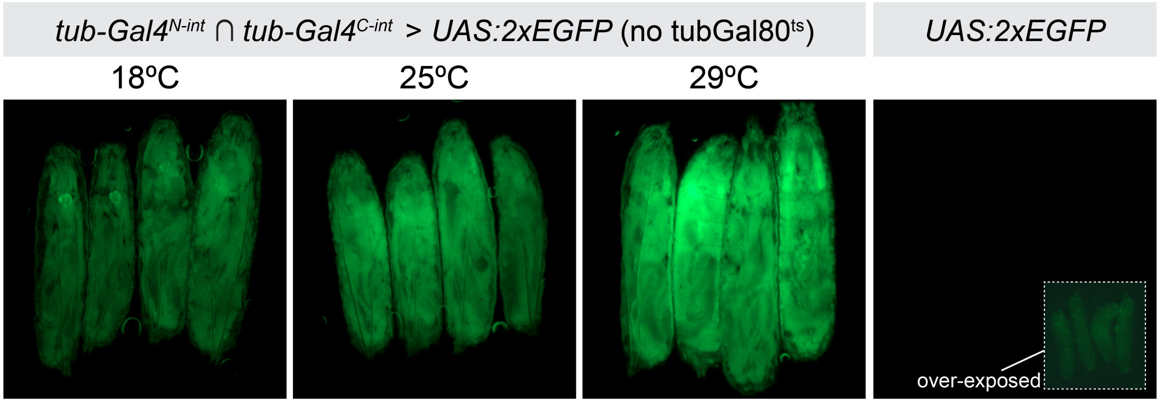

Fig. S2. In the absence of *tub*-Gal80^{ts}, split-intein Gal4 functions at 18ºC, 25ºC, and 29ºC. Larvae were reared at the indicated temperature, and live-imaged at the L3 stage under identical imaging conditions. As with wildtype Gal4 (27), split-intein Gal4 activity is strongest at 29ºC and decreases as the rearing temperature is lowered. The right-most panel indicates negative control, with inset showing an overexposed image to indicate the presence of larva.

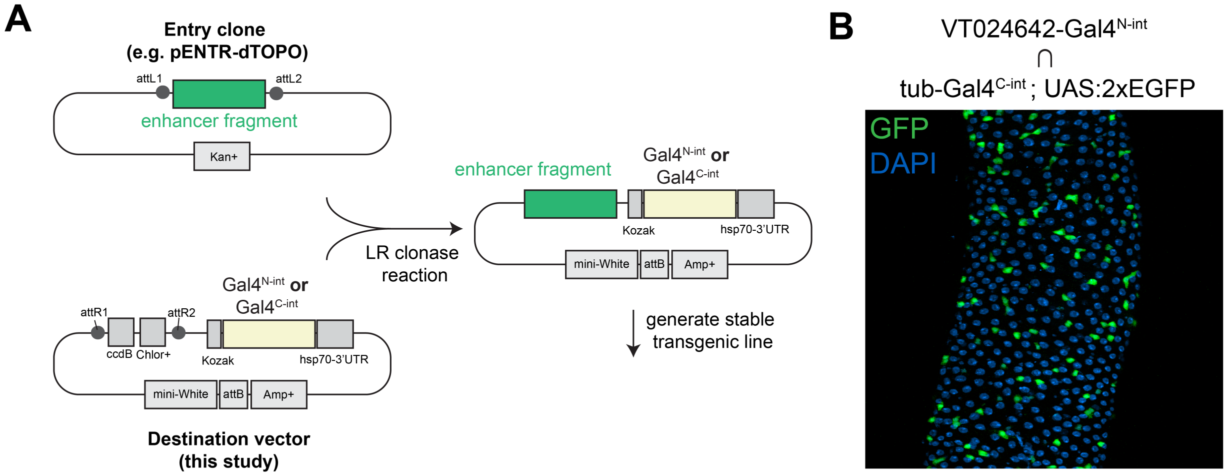

Fig. S3. Enhancer-driven expression of the split-intein system. (A) Gateway LR cloning strategy for cloning split-intein Gal4 components downstream of an enhancer-of-interest. This protocol follows closely the workflow used to generate the split-Gal4 "VT" collection based on 2-3kb enhancer fragments that drive expression in the fly nervous system. (B) Proof of principle for the VT024642 enhancer fragment driving Gal4$^{N\text{-}int}$ in the adult ISCs.

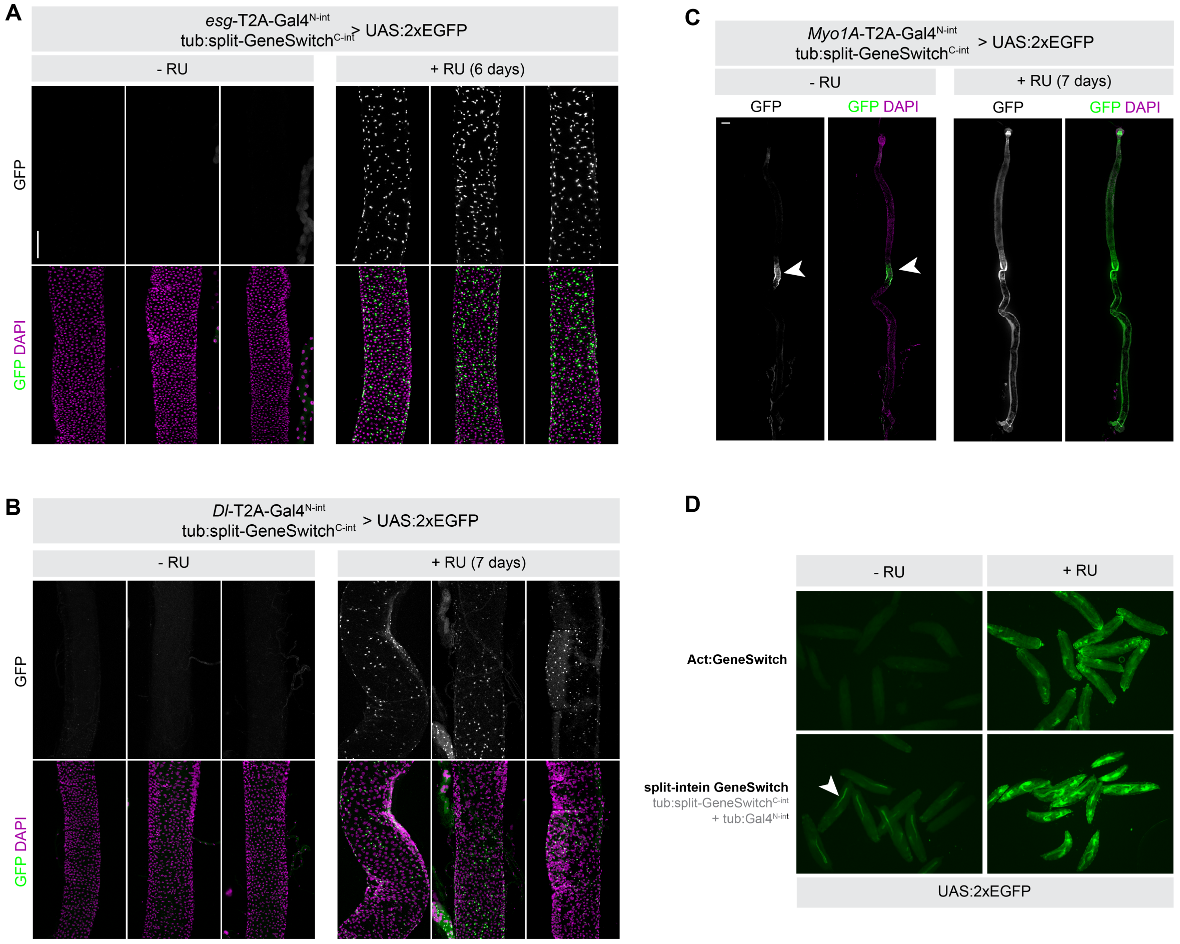

Fig. S4. Characterization of the split-intein GeneSwitch system using multiple drivers. Split-intein GeneSwitch expression in the adult ISCs using either *esg* (A) or *Dl* (B) is non-leaky, and is only observed in the presence of RU. (C) split-intein GeneSwitch expression in enterocytes throughout the adult midgut using *Myo1A*. In the absence of RU (left), expression is observed in a portion of the midgut, whereas RU drives the predicted expression throughout the gut. (D) When split-intein GeneSwitch is expressed ubiquitously using the *tub* promoter, non-RU-dependent expression is visible in portions of the hindgut. *actin*:GeneSwitch (original GeneSwitch) is expressed at lower levels than the *tub* promoter, and does not display this same leakiness. Scale bars = 50μm.

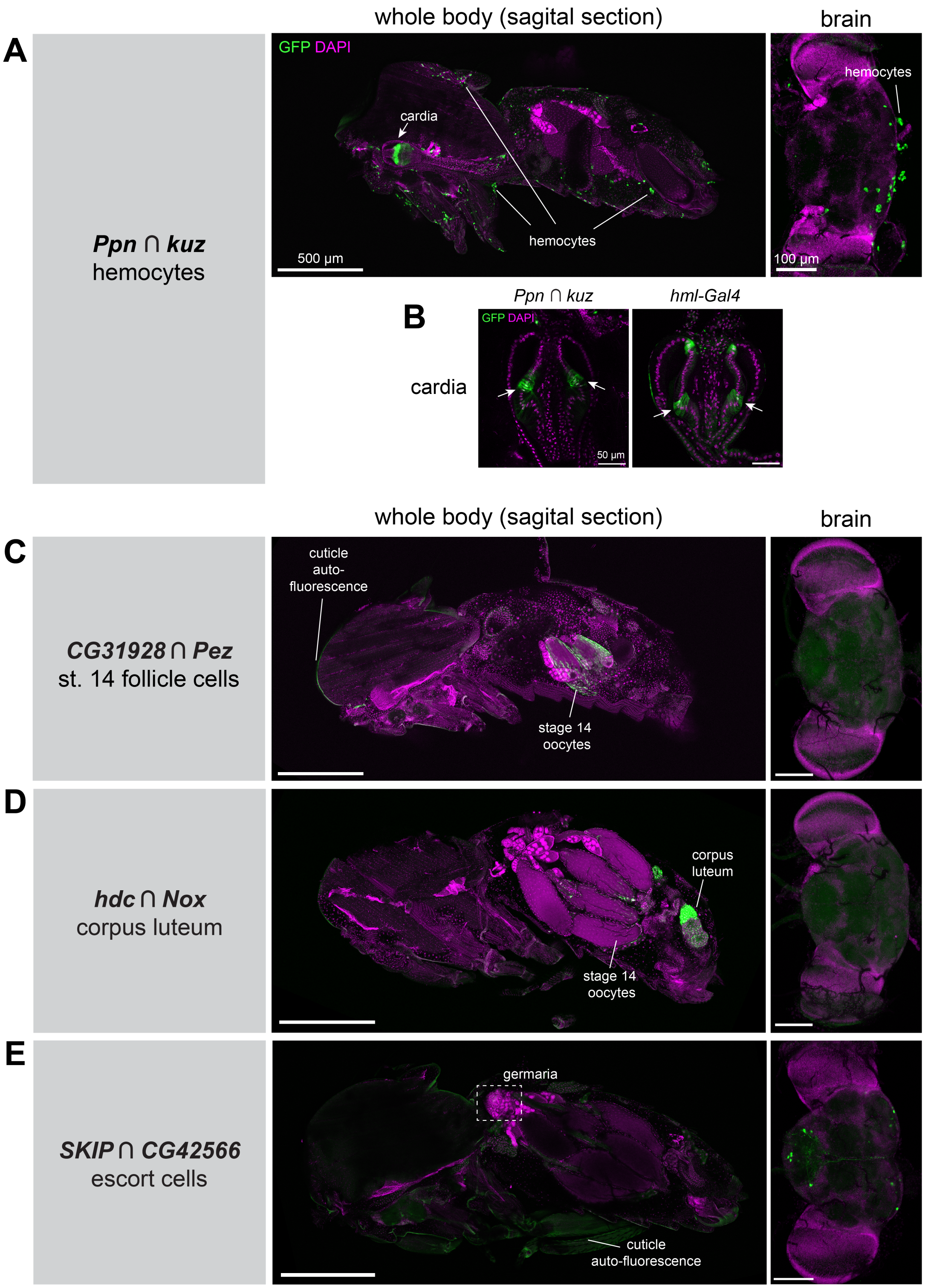

Fig. S5. Whole-body characterization of split-intein Gal4 lines generated from TAB predictions. For each split-intein Gal4 line, UAS:2XEGFP expression is shown in sagittal sections of decapitated adult female flies, as well as in the

adult brain. (A) *Ppn* ∩ *kuz* expression is restricted to circulating adult hemocytes, as well as a band of epithelial cells in the cardia (also known as the proventriculus.) (B) Higher magnification of *Ppn* ∩ *kuz* in the cardia, in comparison with expression driven by the pan-hemocyte marker *hml-Gal4.* Anterior is up. (C-E) Expression of the indicated split-intein Gal4 driver lines, predicted by the TAB algorithm. In sectioned images, anterior is to the left. In brain images, dorsal is to the right.

Purpose (Transgenesis method)	Plasmid map	Cloning strategy

pHD-T2A-split-inteinGal4

Long homology arms
(CRISPR knock-in)

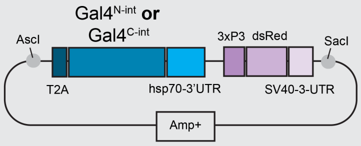

1. Digest with AscI + SacI
2. PCR amplify homology arms w/ overhangs
3. Gibson cloning

- sgRNA co-injected as separate plasmid

Reference: *Bosch et al. 2019*

pDropIn-split-inteinGal4

"Drop-in" cloning
(CRISPR knock-in)

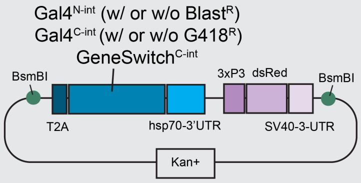

1. Release insert with BsmBI digest
2. Ligate into digested drop-in cassette (pUC57_Kan_gw_OK2)

- sgRNA included on same plasmid

Reference: *Kanca et al. 2019*

pBP-split-inteinGal4-destination

Enhancer driven
(phiC31 integrase transgenesis)

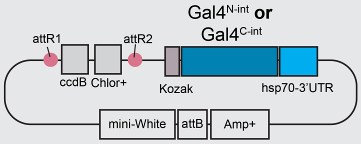

1. Clone enhancer into Gateway donor vector
2. Perform LR Clonase reaction

Reference: *Pfeiffer et al. 2008*

Fig. S6. Plasmid toolkit for generating split-intein Gal4 lines via CRISPR-based knock-in or enhancer-driven. All plasmids are available via AddGene.

Table S1: Transgenic Drosophila lines generated in this study

Line name	Genotype*	sgRNA for knock-in
**Enhancer-driver lines:**		
tub-Gal4[N-int]	w ; P{y[+t7.7]w[+mC]alphaTubulin48B-Gal4-N-int}attP40	N/A
tub-Gal4[C-int]	w ; P{y[+t7.7]w[+mC]alphaTubulin48B-Gal4-C-int}attP40	N/A
tub-GeneSwitch[C-int]	w ; PI{y[+t7.7]w[+mC]alphaTubulin48B-GeneSwitch-C-int}attP40	N/A
VT024642-Gal4[N-int]	w ; P{y[+t7.7]w[+mC]VT024642-Gal4-N-int}attP40	N/A
**T2A knock-in lines ("drop-in" method)**		
esg-T2A-Gal4[N-int]	w ; TI{T2A-Gal4-N-int}esg / CyO	CTCCACCAACATGTCTTCCA
Myo1A-T2A-Gal4[N-int]	w ; TI{T2A-Gal4-N-int}Myo1A / CyO	CCATGGACACTTGGTCGAGG
Delta-T2A-Gal4[C-int]	w ;; TI{T2A-Gal4-C-int}Dl / TM6b	TCGCTGCTGCAGCGGGGAGT
esg-T2A-Gal4DBD	w ; TI{T2A-Gal4DBD}esg / CyO	CTCCACCAACATGTCTTCCA
Myo1A-T2A-Gal4DBD	w ; TI{T2A-Gal4DBD}Myo1A / CyO	CCATGGACACTTGGTCGAGG
Peritrophin-15a-T2A-Gal4[N-int]	w ; TI{T2A-Gal4-N-int}Peritrophin-15a / CyO	TTCGTGGCGCTCCTAAGCAC
CG4830-T2A-Gal4[C-int]	w ;; TI{T2A-Gal4-C-int}CG4830 / TM6b	GTTACCTTGCAGCTACGATG
CG43774-T2A-Gal4[N-int]	w ; TI{T2A-Gal4-N-int}CG43774 / CyO	TACGCGAATCCATTGGCTTG
thetaTry-T2A-Gal4[C-int]	w ; TI{T2A-Gal4-C-int}thetaTry / CyO	GGCACAGTCGGGGTCTCCAA

LManV-T2A-Gal4[N-int]	w ; TI{T2A-Gal4-N-int}LManV / CyO	AAGTCAGCCCAAGCTGTTTG
ninaD-T2A-Gal4[C-int]	w ; TI{T2A-Gal4-C-int}ninaD / CyO	CGGCCTGGGAACCTTTTTCG
Ppn-T2A-Gal4[N-int]	w ;; TI{T2A-Gal4-N-int}Ppn / TM6b	TGGGGCGAACATGTACTTGC
kuz-T2A-Gal4[C-int]	w ; TI{T2A-Gal4-C-int}kuz / CyO	ATAGTTGAGTGTTTCATAGT
CG31928-T2A-Gal4[N-int]	w ; TI{T2A-Gal4-N-int}CG31928 / CyO	ACCGAGTACTATGTTACGGC
Pez-T2A-Gal4[C-int]	w ; TI{T2A-Gal4-C-int}Pez / CyO	GTTCGCCACTACGTGACCAC
hdc-T2A-Gal4[N-int]	w ;; TI{T2A-Gal4-N-int}hdc / TM6b	CCTGAACGAGCTCTCCCTGG
Nox-T2A-Gal4[C-int]	w ; TI{T2A-Gal4-C-int}Nox / CyO	AGGGCGTGGCGGGAATCTCC
SKIP-T2A-Gal4[N-int]	w ;; TI{T2A-Gal4-N-int}SKIP / TM6b	AGCTCTCGGCGTATTGGGCC
CG42566-T2A-Gal4[C-int]	w ; TI{T2A-Gal4-C-int}CG42566 / CyO	TGGCCCGGAACCGTGCATCG
CG13321-T2A-Gal4[N-int] + CG6484-T2A-Gal4[C-int] ; UAS:2xEGFP	w ; TI{T2A-Gal4-N-int}CG13321, TI{T2A-Gal4-C-int}CG6484 / CyO ; UAS:2xEGFP / TM6b	CCAGCTAGGATGGCTCCTGG, ATTGTGTCGGGCGTGCTGTA

*** May be segregating nos:Cas9 on a separate chromosome**

Table S2. Table of genotypes

Figure 2B - Myo1A-Gal4DBD	*y[1] w[*] / w[1118]; P{w[+mC]=Tub-dVP16AD.D}2, P{w[+mC]=UAS-2xEGFP}AH2 / TI{T2A-Gal4DBD}Myo1A ; Dr[1] or TM3, Sb[1]*
Figure 2B - Myo1A-Gal4[N-int]	*w[1118]; P{y[+t7.7]w[+mC]alphaTubulin48B-Gal4-C-int}attP40 / TI{T2A-Gal4-N-int}Myo1A / P{w[+mC]=UAS-2xEGFP}AH3 / +*
Figure 2B - esg-Gal4DBD	*y[1] w[*] / w[1118]; P{w[+mC]=Tub-dVP16AD.D}2, P{w[+mC]=UAS-2xEGFP}AH2 / TI{T2A-Gal4DBD}esg; Dr[1] or TM3, Sb[1]*

Figure 2B - esg-Gal4[N-int]	*w[1118]; P{y[+t7.7]w[+mC]alphaTubulin48B-Gal4-C-int}attP40 / TI{T2A-Gal4-N-int}esg ; P{w[+mC]=UAS-2xEGFP}AH3 / +*
Figure 2C - esg-Gal4[N-int]	*w[1118]; P{y[+t7.7]w[+mC]alphaTubulin48B-Gal4-C-int}attP40 / TI{T2A-Gal4-N-int}esg ; P{w[+mC]=UAS-2xEGFP}AH3 / +*
Figure 2C - Dl-Gal4[C-int]	*w[1118]; P{y[+t7.7]w[+mC]alphaTubulin48B-Gal4-C-int}attP40 / + ; P{w[+mC]=UAS-2xEGFP}AH3 / TI{T2A-Gal4-C-int}Dl*
Figure 2C - esg + Dl	*w[1118]; TI{T2A-Gal4-N-int}esg / P{w[+mC]=UAS-2xEGFP}AH2 ; TI{T2A-Gal4-C-int}Dl / +*
Figure 3B - tub-Gal4 > EGFP	*w[1118] ; P{w[+mC]=tubP-GAL80[ts]}20 / CyO or Sp ; P{w[+mC]=UAS-2xEGFP}AH3/ tub-Gal4*
Figure 3B - tub-split-intein Gal4 > EGFP	*w[1118]; P{y[+t7.7]w[+mC]alphaTubulin48B-Gal4-C-int}attP40 / P{y[+t7.7]w[+mC]alphaTubulin48B-Gal4-N-int}attP40 ;P{w[+mC]=UAS-2xEGFP}AH3 / P{w[+mC]=tubP-GAL80[ts]}ncd[GAL80ts-7]*
Figure 3C - esg-Gal4 > yki	*w[1118] ; esg-Gal4, UAS:GFP, tubGal80[ts] ; P{y[+t7.7] w[+mC]=UAS-yki.S111A.S168A.S250A.V5}attP2*
Figure 3C - esg-split intein Gal4 > yki	*w[1118] ; P{y[+t7.7]w[+mC]alphaTubulin48B-Gal4-C-int}attP40 / TI{T2A-Gal4-N-int}esg ;UAS-2xEGFP , P{w[+mC]=tubP-GAL80[ts]}ncd[GAL80ts-7] / P{y[+t7.7] w[+mC]=UAS-yki.S111A.S168A.S250A.V5}attP2*
Figure 4B - esg-split intein GeneSwitch > UAS:EGFP	*w[1118] ; TI{T2A-Gal4-N-int}esg / PI{y[+t7.7]w[+mC]alphaTubulin48B-GeneSwitch-C-int}attP40 ; P{w[+mC]=UAS-2xEGFP}AH3 / +*
Figure 4B - esg-split intein GeneSwitch > yki	*w[1118] ; TI{T2A-Gal4-N-int}esg / PI{y[+t7.7]w[+mC]alphaTubulin48B-GeneSwitch-C-int}attP40 ; P{w[+mC]=UAS-2xEGFP}AH3 / P{y[+t7.7] w[+mC]=UAS-yki.S111A.S168A.S250A.V5}attP2*
Figure 5A - Peritrophin-15a ∩ CG4830	*y[1] w[*] / w[1118] ; TI{T2A-Gal4-N-int}Peritrophin-15a / + ; TI{T2A-Gal4-C-int}CG4830 / P{w[+mC]=UAS-2xEGFP}AH3 / +*
Figure 5B - CG43774 ∩ thetaTry	*y[1] w[*] / w[1118] ; TI{T2A-Gal4-N-int}CG43774 / TI{T2A-Gal4-C-int}thetaTry / CyO ; P{w[+mC]=UAS-2xEGFP}AH3 / +*

Figure 5C - LManV ∩ ninaD	*y[1] w[*] / w[1118] ; TI{T2A-Gal4-N-int}LManV / TI{T2A-Gal4-C-int}ninaD ; P{w[+mC]=UAS-2xEGFP}AH3 / +*
Figure 6A - Ppn ∩ kuz	*y[1] w[*] / w[1118] ; TI{T2A-Gal4-C-int}kuz / + ; TI{T2A-Gal4-N-int}Ppn / P{w[+mC]=UAS-2xEGFP}AH3*
Figure 6B - CG31928 ∩ Pez	*y[1] w[*] / w[1118] ; TI{T2A-Gal4-N-int}CG31928 / TI{T2A-Gal4-C-int}Pez ; P{w[+mC]=UAS-2xEGFP}AH3 / +*
Figure 6C - hdc ∩ Nox	*y[1] w[*] / w[1118] ; TI{T2A-Gal4-C-int}Nox / + ; TI{T2A-Gal4-N-int}hdc / P{w[+mC]=UAS-2xEGFP}AH3*
Figure 6D - SKIP ∩ CG42566	*y[1] w[*] / w[1118] ; TI{T2A-Gal4-C-int}CG42566 / + ; TI{T2A-Gal4-N-int}SKIP / P{w[+mC]=UAS-2xEGFP}AH3*
Figure 7A & B - CG6484 ∩ CG13321	*w[1118] ; TI{T2A-Gal4-N-int}CG13321, TI{T2A-Gal4-C-int}CG6484 / CyO ; P{w[+mC]=UAS-2xEGFP}AH3 / +*
Figure S2 - VT02642-Gal4[N-int]	*w[1118] ; P{y[+t7.7]w[+mC]VT024642-Gal4-N-int}attP40 / P{y[+t7.7]w[+mC]alphaTubulin48B-Gal4-C-int}attP40 ; P{w[+mC]=UAS-2xEGFP}AH3 / +*
Figure S3 - esg split-intein GeneSwitch	*w[1118] ; TI{T2A-Gal4-N-int}esg ;UAS-2xEGFP / w ; PI{y[+t7.7]w[+mC]alphaTubulin48B-GeneSwitch-C-int}attP40 ; P{w[+mC]=UAS-2xEGFP}AH3*
Figure S3 - Dl split-intein GeneSwitch	*w[1118] ; PI{y[+t7.7]w[+mC]alphaTubulin48B-GeneSwitch-C-int}attP40 ; P{w[+mC]=UAS-2xEGFP}AH3 / TI{T2A-Gal4-C-int}Dl*
Figure S3 - Myo1A split-intein GeneSwitch	*w[1118] ; PI{y[+t7.7]w[+mC]alphaTubulin48B-GeneSwitch-C-int}attP40 / TI{T2A-Gal4-N-int}Myo1A ; P{w[+mC]=UAS-2xEGFP}AH3 / +*
Figure S4 - Act:GeneSwitch	*w[1118] ;; actin:GeneSwitch / P{w[+mC]=UAS-2xEGFP}AH3*
Figure S4 - tub:split intein GeneSwitch	*w[1118] / y[1] w[*] or y[1] w[*] ; PI{y[+t7.7]w[+mC]alphaTubulin48B-GeneSwitch-C-int}attP40 / P{y[+t7.7]w[+mC]alphaTubulin48B-Gal4-N-int}attP40 ; P{w[+mC]=UAS-2xEGFP}AH3 / +*

SI References

1. H. Iwai, S. Züger, J. Jin, P.-H. Tam, Highly efficient protein trans-splicing by a naturally split DnaE intein from Nostoc punctiforme. *Febs Lett* **580**, 1853–1858 (2006).
2. H. Luan, N. C. Peabody, C. R. Vinson, B. H. White, Refined Spatial Manipulation of *Neuron*al Function by Combinatorial Restriction of Transgene Expression. Neuron **52**, 425–436 (2006).
3. P. Carvajal-Vallejos, R. Pallissé, H. D. Mootz, S. R. Schmidt, Unprecedented Rates and Efficiencies Revealed for New Natural Split Inteins from Metagenomic Sources*. *J Biol Chem* **287**, 28686–28696 (2012).
4. A. J. Stevens, *et al.*, A promiscuous split intein with expanded protein engineering applications. *Proc National Acad Sci* **114**, 8538–8543 (2017).
5. J. Xu, et al., Protein visualization and manipulation in Drosophila through the use of epitope tags recognized by nanobodies. *Elife* **11**, e74326 (2022).
6. J. A. Bosch, R. Colbeth, J. Zirin, N. Perrimon, Gene Knock-Ins in Drosophila Using Homology-Independent Insertion of Universal Donor Plasmids. *Genetics* **214**, 75–**8**9 (2019).
7. O. Kanca, et al., An efficient CRISPR-based strategy to insert small and large fragments of DNA using short homology arms. Elife 8, e51539 (2019).
8. F. Port, H.-M. Chen, T. Lee, S. L. Bullock, Optimized CRISPR/Cas tools for efficient germline and somatic genome engineering in Drosophila. *Proceedings of the National Academy of Sciences of the United States of America* **111**, E2967-76 (2014).
9. B. Ewen-Campen, T. Comyn, E. Vogt, N. Perrimon, No Evidence that Wnt Ligands Are Required for Planar Cell Polarity in Drosophila. *Cell Reports* **32**, 108121 (2020).
10. O. Kanca, et al., An expanded toolkit for Drosophila gene tagging using synthesized homology donor constructs for CRISPR-mediated homologous recombination. Elife 11, e76077 (2022).
11. N. Matinyan, et al., Multiplexed drug-based selection and counterselection genetic manipulations in Drosophila. Cell Reports **36**, 109700 (2021).
12. É. Kurucz, et al., Definition of Drosophila Hemocyte Subsets by Cell-Type Specific Antigens. *Acta Biol Hung* **58**, 95–111 (2007).
